# Root traits and root biomass allocation impact how wheat genotypes respond to organic amendments and earthworms

**DOI:** 10.1371/journal.pone.0200646

**Published:** 2018-07-24

**Authors:** Junaidi Junaidi, Cynthia M. Kallenbach, Patrick F. Byrne, Steven J. Fonte

**Affiliations:** 1 Soil and Crop Sciences Department, Colorado State University, Fort Collins, CO, United States of America; 2 Indonesian Rubber Research Institute, Bogor, Jawa Barat, Indonesia; Università Politecnica delle Marche, ITALY

## Abstract

Plant-soil biological interactions are increasingly recognized as a key feature of agroecosystems, promoting both crop and soil health. However, the effectiveness of plant-soil synergies is likely modulated by both root system characteristics and soil management impacts on soil biological communities. To successfully manage for plant-soil interactions, we need to better understand how crops respond to changes in soil management, especially in terms of belowground investment. Specifically, crop genotypes that exhibit reduced plasticity in root growth and investment may not be able to take full advantage of changes in soil biological activity associated with soil health promoting practices. We hypothesized that genotypes with greater belowground investment respond more, in terms of plant growth and crop nitrogen (N) uptake, to compost and earthworm additions, agronomic factors commonly associated with soil health. We evaluated four spring wheat (*Triticum aestivum*) genotypes with distinct breeding and environmental histories, and one progenitor of wheat (*Aegilops tauschii*) under low soil fertility conditions in the greenhouse for differences in belowground root biomass and architecture. We then determined how these belowground traits influenced genotype response to additions of compost and earthworms. Measurements included plant growth, biomass, grain yield, root characteristics, plant N uptake, and soil N. Overall, in unamended soils, genotypes differed in above and belowground phenotypic traits. In general, *Ae*. *tauschii* had three times greater root: shoot (R:S) ratio, root length, and root biomass relative to wheat genotypes. We found that genotypes with higher R:S ratios responded more positively to compost additions compared to those with lower R:S ratios, particularly in terms of plant aboveground biomass, N uptake and soil N-cycling, and also exhibited greater plasticity in root morphology. Consequently, while higher R:S genotypes had relatively poorer yields in unamended soils, they outperformed lower R:S genotypes in total seed weight under compost treatments. Our findings suggest that genotypes with greater belowground investment may be better able to take advantage of soil health promoting practices, such as the use of organic amendments. These results highlight the need to consider soil management practices (and associated biological communities) in parallel with root phenotypic plasticity when evaluating wheat lines for improvements in plant-soil synergies.

## 1. Introduction

Soil organisms regulate many belowground functions that benefit plants, including organic matter decomposition, nutrient transformations, maintenance and formation of soil structure, and biocontrol of soil-borne plant pathogens [1]. In turn, plants provide primary substrates for supporting an active and diverse soil food web via organic inputs (e.g. roots, aboveground residues, and root exudates) [2,3,4]. Such plant-soil feedbacks have received considerable attention in recent years and are thought to hold immense potential for improving agricultural sustainability and resilience [5,6,7,8]. In particular, greater belowground carbon (C) allocation and root traits such as root length and diameter, are key factors modulating plant-soil interactions that influence nutrient cycling and other fundamental soil processes [7]. In cropping systems that depend on organic nutrient sources (e.g., compost, manure, cover crops), these rhizosphere biological interactions are especially important, as crop nutrient availability relies largely on the soil biological community and its activity [9].

The challenge is that crop species and even genotypes have distinct rooting systems, affecting the ability of crops to facilitate plant-soil interactions [10,11]. Moreover, it is unclear how root phenotypic differences interact with changes in soil management. Plant root systems are recognized as a major selective force on soil biological communities [12,13,14,15], and thus selecting genotypes with greater root inputs may offer a feasible approach for developing cropping systems that can enhance beneficial plant-soil interactions [7].

In response to widespread concern over detrimental impacts of intensive, high-input agriculture on soil health and climate change, e.g. [16,17], soil health promoting agricultural practices are on the rise globally [18,19, 20]. However, missing from these efforts is the development of crop genotypes with appropriate root traits that encourage crop-soil synergies and support processes such as biologically mediated nutrient availability [14, 21, 22]. Though significant crop genotypic advances have been made on disease resistance and effective use of resources, few have specifically targeted belowground phenotypic traits that support plant-soil interactions [22]. The onset of intensive agriculture in the last century coincided with breeding efforts focused on producing high-yielding genotypes under high mineral nitrogen (N) conditions [22, 23, 24]. Moreover, intensive agricultural practices (e.g. frequent application of pesticides, inorganic fertilizers and tillage) can negatively impact soil biological communities and potentially lead to a decoupling of plant and soil communities [25]. Thus, even if genotypes exhibit enhanced root allocation, the soil microbes and fauna that constitute the other half of these interactions, may still be inhibited by the agricultural practices in place.

Rhizosphere interactions can easily be disrupted or promoted, as microbes and fauna are highly sensitive to nutrient and other abiotic modifications resulting from management interventions such as tillage, fertilization or organic amendments [11, 26, 27, 28]. Several soil health promoting practices are well known to enhance soil biological activity and thus promote N mineralization of organic resources. Mineralization of organic matter inputs can be a significant source of plant available mineral N across a diversity of cropping systems and is especially important in organic and low input systems [29]. Low C:N compost or manure amendments often stimulate soil biological activity [30], and this can improve a range of soil physical properties [31], increase gross N mineralization, and enhance root growth [32]. Similarly, higher abundances of certain soil fauna, such as earthworms, can improve soil structure and aggregation [33], as well as increase plant N availability, either directly through organic matter and residue turnover [34, 35] or through stimulation of the microbial community [36]. However, genotypes bred primarily for yield traits may not be able to benefit fully from these practices that enhance biologically-mediated N cycling if belowground C allocation is subsequently reduced, limiting interactions with key soil organisms [37]. Thus, to improve the benefits of soil health promoting practices, such as the use of organic amendments, there is a need to evaluate crop phenotypic plasticity along with changes in belowground biological activities and the soil environment.

A comparison of diverse crop genotypes that includes recently released genotypes, landraces, and wild accessions could serve as a useful framework for examining these interactions between phenotypic variability and soil health improvements [37]. Based on co-occurring historical changes in breeding environments and objectives, a gradient in belowground C allocation may exist along a domestication continuum, effecting rhizosphere interactions [38,39,40]. Several studies have now demonstrated that ancestral and early crop landraces exhibit greater mycorrhizal dependencies and responses compared to modern varieties [41,42,43,44]. One hypothesis for this is that crops genetically more similar to their wild ancestors, or selected for under systems more similar to natural environments, may have maintained traits such as greater root length or root: shoot (R:S) ratios, to facilitate plant-soil feedbacks [6,39,42].

Wheat, representing about 20% of the human food supply [45], has a long history of human selection and intentional breeding and thus is a relevant crop species for comparisons of genotypes, especially across a historical breeding gradient. Following the hybridization of the wild diploid species *Aegilops tauschii* with primitive tetraploid wheat (*Triticum dicoccoides*) to form hexaploid bread wheat, early farmers developed landraces specific to local agroecosystem conditions. As food demand increased, selection activities were followed to obtain genotypes with high yield potential and tolerance to diseases, potentially affecting root biomass allocation and morphology [23,38,46]. Indeed, evidence of wheat domestication effects on root systems has previously been shown for root exudate profiles, architecture and biomass [38,47,48].

Here we examine how spring wheat genotypes representing varied breeding histories, and a wild accession, express diverse phenotypic traits related to root biomass and morphology under controlled, nutrient limited conditions. We examine how differences in root traits impact genotype growth responses to soil health improvements, achieved via additions of organic matter and key soil fauna. We hypothesize that genotypes exhibiting greater belowground C allocation will exhibit greater response (in growth, yield, and plant and soil N dynamics) to composted manure and earthworm additions.

## 2. Materials and methods

### 2.1 Experimental design and implementation

This study was carried out in a greenhouse at Colorado State University, Fort Collins, CO, USA between June and November, 2016. The study utilized a full factorial complete randomized design (CRD) with three factors: genotype (5 levels), compost additions (2 levels), and earthworm additions (2 levels). Different genotypes of spring wheat (*Triticum aestivum*) and its progenitor, *Aegilops tauschii*, were grown to evaluate the interactive impacts of phenotypic trait variations, earthworms and compost on wheat growth parameters. Different genotypes of spring wheat (*Triticum aestivum*) and one of its progenitor species (*Aegilops tauschii*, the D genome donor of wheat) were grown to evaluate the interactive impacts of phenotypic trait variations, earthworms and compost on wheat growth parameters. The *Ae*. *tauschii* accession (TA2374 [49]) was originally collected in Pakistan in 1955, and seeds were provided by the Wheat Genetics Resource Center, Manhattan, Kansas. The two older genotypes of spring wheat were the obsolete variety ‘Gypsum’ and the landrace ‘Red Fife’. Gypsum was bred in Colorado, USA [50] and was released in 1912, while the landrace Red Fife was developed as early as 1842, originating in Eastern Europe and later becoming an important variety in North America [51, 52]. The Gypsum and Red Fife seeds were obtained from the USDA-ARS National Small Grains Collection, Aberdeen, Idaho. Two recently released genotypes from this century, Scholar Rht2M and Scholar Rht2W (referred to here as Rht2M and Rht2W), originated in Montana, USA and were obtained from Montana State University, Bozeman, Montana [53,54]. The recently released genotypes are near-isogenic to each other; Scholar Rht2W contains the tall allele *Rht-D1a* at the *Rht-D1* locus, while Scholar Rht2M contains the widely deployed semi-dwarf allele *Rht-D1b*. For simplicity throughout the results and discussion we refer to Gypsum and Red Fife as *older varieties* and the recently released wheat genotypes, Rht2W and Rht2W, as *modern* relative to the older varieties and the wild accession, *Ae*. *tauschii*.

Plants were grown in 24.5 x 3.5 x 38.0 cm deep rectangular plastic boxes, hereafter called rootboxes [55]. The rootboxes were angled at 25° from vertical and had one side that could be opened for root observations and rhizosphere soil sampling. Each box was filled with a soil-sand media (~ 4700 g box^-1^) and packed by gentle shaking. The soil was collected (0–20 cm depth) from the Agricultural Research, Development and Education Center (ARDEC) located 6.5 km north of Fort Collins, Colorado (40°39'10.3"N 104°59'46.6"W). The soil is a clay loam, mixed, superactive, mesic Aridic Haplustalfs soil, has a pH of 8.7, and contains 1.16% total C, 0.1% 1.5 g total soluble N kg^-^ soil, total N, 17 ppm Olsen P, and 185 ppm K [56]. The soil was first sieved (4.0 mm), air-dried, and then mixed with sand (1:1 volume) to avoid soil compaction and maintain drainage. Prior to seeding, we mixed in 1.0 g of granular NPK 16-16-16 fertilizer (6.25% ammonium, 9.75% urea) to 5–6 cm depth in each box. Direct seeding at 2–3 cm depth was conducted for landraces and modern genotypes by planting two seeds per rootbox, while *Ae*. *tauschii* seedlings were transplanted following a 10-day pre-germination cold treatment (4°C). Plants were thinned to one plant per box two weeks after planting for all treatments. All plants were grown under a 16 hr growing day, at approximately 21° C and 50% humidity until harvest, and at approximately 70% soil water holding capacity, amounting to 174.76 g of water kg^-^ soil media at 70% of field capacity. When wheat plants reached the mealy ripe stage (Zadok’s code 85), soil moisture was adjusted to 60% to match reduction in plant water use. Pests, mainly aphids, were controlled as needed using light insecticide application.

The compost treatments included either soils amended with composted manure (from the Aurora Dairy in Greeley, Colorado) or soils without compost. Compost amendments provided an additional 22, 7, and 36 mg of NPK kg^-^ soil. The compost had a C:N of 41.29 and pH of 8.82. Compost macronutrient composition was 8.56, 2.74, and 13.63 g NPK kg^-^ dry matter. The compost micronutrient composition was 68.74 ppm Zn; 2475.2 ppm Fe; 128.57 ppm Mn; 57.16 ppm Cu; and 24.56 ppm B. Composted manure was passed through a 4.0 mm sieve and applied at a rate of 300 ml per box (195.6 g fresh wt.) by thoroughly mixing with the soil-sand mixture. For the earthworm treatment, each box either received two mature individuals of the earthworm *Aporrectodea caliginosa* per box (~ 1.7 g total fresh biomass) or no earthworm addition. All individuals of *A*. *caliginosa*, a commonly occurring species in Colorado [57], were collected from ARDEC within the top 30 cm of soil from a nearby field to where soil was collected. Five replications were established for each of the twenty treatments, leading to 100 experimental units (plants) in total.

### 2.2 Plant growth and yield assessment

Heading date and number of tillers were recorded for each plant in the study. Following harvest, we measured aboveground components and root biomass, root morphology characteristics, and N content of the grain and vegetative aboveground biomass (details provided below). Heading date was recorded when the spike emerged fully above the flag leaf. Number of tillers was counted one day before harvest. We determined physiological maturity to have occurred when the peduncle of the first spike had turned completely yellow. Harvest was carried out separately for each genotype when 50% of the plants in treatments of the same genotype reached physiological maturity. We harvested total aboveground biomass by cutting stems just above the soil surface and drying at 60^º^ C. Total biomass is reported as the sum of aboveground components and roots. Number of seeds was counted after threshing the spikelets manually and then yield was determined as the total weight of the seeds per plant (after removing seeds from the enclosing capsules in the case of *Ae*. *tauschii*). We calculated the average weight per seed by dividing yield by the number of seeds per plant. Harvest index was determined as the yield divided by total aboveground biomass.

Following aboveground biomass collection at harvest, we first examined the soil for earthworm presence and then rinsed the soil with water to separate coarse roots from the soil. The fine roots were separated from soil using an elutriator (Standard Industries Inc., Fargo, ND USA). We determined root morphology characteristics using a root scanning procedure following [58] on a ScanMaker 9800XL (Microtek International Inc., Santa Fe Springs, CA USA) with gray scale scanning type and 600 dpi resolution. To improve image contrast and root length estimates, we submerged all roots in staining liquid (Organic Neutral Red Stain) before scanning [59]. Root morphology was evaluated using WinRhizo software (Regent Instrument Inc., Quebec, Canada) and measurements included root length, root surface area, root volume, and average root diameter. For each diameter class (0.00–0.25, 0.25–0.50, 0.50–0.75, 0.75–1.00, and > 1.00 mm), root length, surface area, and volume were obtained. We calculated specific root length as root length relative to total root biomass (m g^-1^). After scanning, roots were rinsed, oven-dried, and weighed to determine total belowground biomass.

### 2.3 Plant and soil nitrogen analyses

Grain and aboveground biomass components (including stems, leaves, and chaff) were ground and analyzed separately for total N concentration using a TruSpec® CN (LECO Corporation, Saint Joseph, MI USA). Aboveground N uptake was calculated by multiplying N concentration by dry shoot biomass.

At tillering, heading, and harvest (approximate Zadoks stages 22, 58, and 92; [60]), rhizosphere soil was collected from each rhizobox by sampling at 10 separate locations within 0.5 cm of root structures. Soils were sampled from all treatments (*n* = 100). However, for laboratory analyses from soils collected at wheat harvest sampling, earthworm treatments (*n* = 50) were excluded due to their poor survival. Sampled soils were composited and stored at 4°C for less than one week for subsequent soil nitrate (NO_3_^-^), ammonium (NH_4_^+^) and microbial biomass C (MBC) and N (MBN) analyses. Soil NO_3_^-^and NH_4_^+^ concentrations (μg g^-1^ dry soil) were determined on 0.5M K_2_SO_4_ extract on a Alpkem Flow Solution IV Automated system (O.I. Analytical, College Station, TX) and soil MBC and MBN were determined after a chloroform fumigation extraction procedure (0.5M K_2_SO_4_) [61] on a Shimadzu TOC-L with a TNM-L Total Nitrogen Module (Shimadzu Scientific Instruments, Inc.).

### 2.4 Data analysis

Analyses of plant and soil variables were performed using R Statistical Software version 3.2.5 (R Development Core Team) in R Studio (version 1.0.136) environment. Three-way ANOVA with a CRD model was run for all plant variables as well as two- and three-way interactions. To satisfy ANOVA assumptions, variables were square root transformed as needed (heading date, aboveground biomass, root length, average root diameter, harvest index, and vegetative biomass N concentration) or ln transformed (total aboveground N uptake). For several variables where the wild accession, *Ae*. *tauschii*, presented extreme values and we obtained significant genotype effects, we reran the three-way ANOVA excluding *Ae*. *tauschii* to determine if genotype effects still persisted among the domesticated wheat only. However, all pairwise comparisons and ANOVAs presented are from analyses where all 5 levels of genotype are included. In cases where genotype effects were strictly driven by *Ae*. *tauschii*, we state as such within the text. Treatment effects on soil parameters NO_3_^-^, NH_4_^+^, MBC, and MBN were determined using a three-way mixed-effects, structured, repeated- measures ANOVA model (earthworm treatments excluded), with time, compost, and genotype as the three fixed factor and rhizobox as the repeated measure. We evaluated the relationships between plant growth and soil N cycling parameters from wheat harvest sampling using Pearson’s correlation coefficients (ρ). Least squares means were estimated using lsmeans function and compared using Tukey method with α = 0.05. Summary statistics are given in original units. All data is available within Supporting Information, S1 Table.

## 3. Results

Of the 100 experimental units, 95 plants were used for analyses, as 5 plants did not survive or were severely stunted (2 *Ae*. *tauschii*; 2 Gypsum; 1 Rht2W). Most of the earthworms did not survive until the end of the experiment. However, evidence of their activities was observed throughout the first month and at harvest (via surface casting), and significant burrowing and casting activity was noted in nearly all earthworm addition treatments. Thus, the earthworm factor was included in all plant and root analyses.

### 3.1 Plant growth and biomass

Genotype impacted (p < 0.001) belowground C allocation (root biomass and R:S ratio) and aboveground plant growth (aboveground biomass, heading date and number of tillers) (Table 1, Table 2, S1 Fig). We observed a gradient of belowground C allocation based on root biomass and R:S, where *Ae*. *tauschii* > Gypsum> Rht2M = Rht2W> Red Fife. However, this gradient was not distinctly linear with genotype release date. *Ae*. *tauschii* took nearly twice as long to reach heading, had more tillers, greater root biomass, and higher R:S ratio compared to the four *Triticum* genotypes (Table 1, S1 Fig). Relative to the other wheat genotypes, Gypsum generally had a longer time to reach heading, had more tillers, higher above- and belowground biomass, and higher R:S ratio. Compost also had a main effect (across all genotypes) on the heading date and number of tillers (p < 0.001) as well as root biomass (p = 0.015) (Table 1, S1 Fig). Earthworms delayed heading date by 2.7% on average (p = 0.044) but did not influence any of the other plant growth or biomass variables.

**Table 1:** Means and ANOVA results for belowground biomass and root architecture traits for *Ae*. *tauschii* and four spring wheat genotypes grown in the greenhouse under differing soil treatments. Standard errors are presented to the right of each mean.

Treatment^a^^b^	Root biomass (g plant^-1^)	Root:shoot ratio	Root length (m plant^-1^)	Root angle (°)	Root surface area (cm^2^)	Mean root diameter (mm)
*Ae*. *tauschii*						
NC-NE	1.62 ± 0.16	0.140 ± 0.013	378.2 ± 49.6	83 ± 6.4	2206 ± 295	0.96 ± 0.13
NC-E	1.14 ± 0.10	0.121 ± 0.010	228.7 ± 26.6	79 ± 8.0	1394 ± 160	0.73 ± 0.04
C-NE	2.85 ± 0.60	0.163 ± 0.016	681.5 ± 191.6	77 ± 2.0	4054 ± 1126	1.40 ± 0.24
C-E	4.27 ± 2.72	0.212 ± 0.085	616.4 ± 268.2	72 ± 8.5	3636 ± 1173	1.08 ± 0.30
Gypsum						
NC-NE	0.57 ± 0.12	0.050 ± 0.005	130.9 ± 26.9	79 ± 1.6	725 ± 133	0.53 ± 0.01
NC-E	0.56 ± 0.09	0.052 ± 0.005	125.1 ± 20.3	80 ± 3.4	716 ± 117	0.54 ± 0.02
C-NE	0.68 ± 0.07	0.052 ± 0.001	154.0 ± 23.3	81 ± 2.9	845 ± 107	0.53 ± 0.01
C-E	0.80 ± 0.09	0.069 ± 0.005	219.7 ± 45.9	80 ± 4.5	1148 ± 225	0.53 ± 0.03
Rht2M						
NC-NE	0.35 ± 0.06	0.043 ± 0.003	78.4 ± 16.6	51 ± 1.5	453 ± 82	0.56 ± 0.02
NC-E	0.41 ± 0.06	0.040 ± 0.002	78.1 ± 12.5	64 ± 4.5	457 ± 65	0.56 ± 0.02
C-NE	0.39 ± 0.04	0.038 ± 0.001	90.5 ± 11.8	64 ± 1.6	493 ± 53	0.53 ± 0.02
C-E	0.36 ± 0.10	0.034 ± 0.004	100.2 ± 32.2	61 ± 3.6	517 ± 171	0.37 ± 0.05
Rht2W						
NC-NE	0.47 ± 0.05	0.041 ± 0.003	100.0 ± 12.7	57 ± 3.7	570 ± 68	0.55 ± 0.02
NC-E	0.31 ± 0.03	0.036 ± 0.003	66.7 ± 10.9	66 ± 4.5	384 ± 61	0.55 ± 0.02
C-NE	0.39 ± 0.06	0.041 ± 0.006	113.2 ± 22.9	63 ± 3.2	625 ± 127	0.53 ± 0.03
C-E	0.28 ± 0.06	0.037 ± 0.002	66.6 ± 29.7	75 ± 4.7	371 ± 149	0.29 ± 0.06
Red Fife						
NC-NE	0.31 ± 0.02	0.031 ± 0.002	76.2 ± 8.7	71 ± 3.0	433 ± 58	0.53 ± 0.01
NC-E	0.46 ± 0.04	0.041 ± 0.003	120.0 ± 8.6	70 ± 3.4	664 ± 56	0.52 ± 0.01
C-NE	0.30 ± 0.08	0.038 ± 0.006	76.1 ± 28.1	66 ± 6.2	426 ± 146	0.56 ± 0.02
C-E	0.27 ± 0.12	0.031 ± 0.006	79.1 ± 39.6	74 ± 6.5	421 ± 191	0.31 ± 0.05
G	***	***	***	***	***	***
C	*	ns	*	ns	**	ns
E	ns	ns	ns	ns	ns	***
G x C	**	*	**	ns	***	***
G x E	ns	ns	ns	ns	ns	ns
C x E	ns	ns	ns	ns	ns	***
G x C x E	ns	ns	ns	ns	ns	ns

^a^ No compost or earthworms added (NC-NE); no compost, but earthworms added (NC-E); compost added, no earthworms (C-NE); both compost and earthworms added (C-E).

^b^ Significance indicated (*, p < 0.05; **, p < 0.01; ***, p < 0.001; ns, not significant) for all experimental factors and interactions (G, genotype; C, compost; E, earthworm); *n* = 5.

**Table 2:** Means and ANOVA results for aboveground production variables for *Ae*. *tauschii* and four spring wheat genotypes grown in the greenhouse under differing soil treatments. Standard errors are presented to the right of each mean.

Treatment^a^^b^	Aboveground biomass (g plant^-1^)	Number of seeds (seeds plant^-1^)	Yield (g plant^-1^)	Average weight per seed (mg seed^-1^)	Harvest index
*Ae*. *tauschii*					
NC-NE	11.5 ± 0.1	252.8 ± 5.2	1.11 ± 0.04	4.38 ± 0.17	0.096 ± 0.004
NC-E	9.5 ± 0.4	154.0 ± 28.5	0.66 ± 0.15	4.04 ± 0.37	0.067 ± 0.014
C-NE	16.9 ± 2.2	275.8 ± 42.0	1.25 ± 0.29	4.37 ± 0.37	0.076 ± 0.014
C-E	16.8 ± 4.5	206.0 ± 118.3	1.05 ± 0.86	3.40 ± 1.49	0.046 ± 0.032
Gypsum					
NC-NE	10.9 ± 1.4	116.5 ± 15.5	2.70 ± 0.43	22.91 ± 1.82	0.243 ± 0.016
NC-E	10.5 ± 0.9	101.8 ± 27.1	2.25 ± 0.63	22.04 ± 1.13	0.204 ± 0.048
C-NE	13.0 ± 1.4	152.8 ± 21.3	3.35 ± 0.36	22.72 ± 2.46	0.259 ± 0.013
C-E	11.5 ± 0.7	98.4 ± 11.4	1.96 ± 0.19	20.35 ± 1.42	0.170 ± 0.011
Rht2M					
NC-NE	8.2 ± 1.5	144.0 ± 28.4	2.71 ± 0.59	18.36 ± 1.00	0.318 ± 0.021
NC-E	10.3 ± 0.8	167.2 ± 11.5	3.81 ± 0.46	23.12 ± 3.06	0.369 ± 0.036
C-NE	10.4 ± 1.3	193.6 ± 32.9	3.52 ± 0.63	18.17 ± 0.61	0.333 ± 0.022
C-E	9.7 ± 1.8	167.8 ± 37.9	3.06 ± 0.83	17.31 ± 1.42	0.296 ± 0.034
Rht2W					
NC-NE	11.4 ± 0.6	169.6 ± 5.5	3.47 ± 0.32	20.63 ± 2.30	0.303 ± 0.015
NC-E	8.6 ± 0.5	124.8 ± 13.8	2.78 ± 0.36	22.46 ± 2.63	0.320 ± 0.034
C-NE	9.6 ± 1.2	145.5 ± 21.1	2.99 ± 0.50	20.57 ± 1.88	0.309 ± 0.029
C-E	7.6 ± 1.6	93.6 ± 39.2	1.91 ± 0.84	18.17 ± 1.70	0.200 ± 0.068
Red Fife					
NC-NE	10.0 ± 0.3	126.8 ± 9.9	2.90 ± 0.23	23.04 ± 1.22	0.290 ± 0.021
NC-E	11.2 ± 1.0	136.8 ± 15.2	3.36 ± 0.56	24.35 ± 1.79	0.295 ± 0.024
C-NE	7.4 ± 0.9	82.0 ± 9.6	1.95 ± 0.29	23.73 ± 1.51	0.264 ± 0.022
C-E	7.5 ± 2.0	104.2 ± 35.8	2.00 ± 0.57	20.45 ± 1.60	0.254 ± 0.028
G	***	***	***	***	***
C	ns	ns	ns	*	*
E	ns	**	ns	ns	**
G x C	***	ns	ns	ns	ns
G x E	ns	ns	ns	ns	ns
C x E	ns	ns	ns	*	*
G x C x E	ns	ns	ns	ns	ns

^a^ No compost or earthworms added (NC-NE); no compost, but earthworms added (NC-E); compost added, no earthworms (C-NE); both compost and earthworms added (C-E).

^b^ Significance indicated (*, p < 0.05; **, p < 0.01; ***, p < 0.001; ns, not significant) for all experimental factors and interactions (G, genotype; C, compost; E, earthworm); *n* = 5.

**Fig 1:**
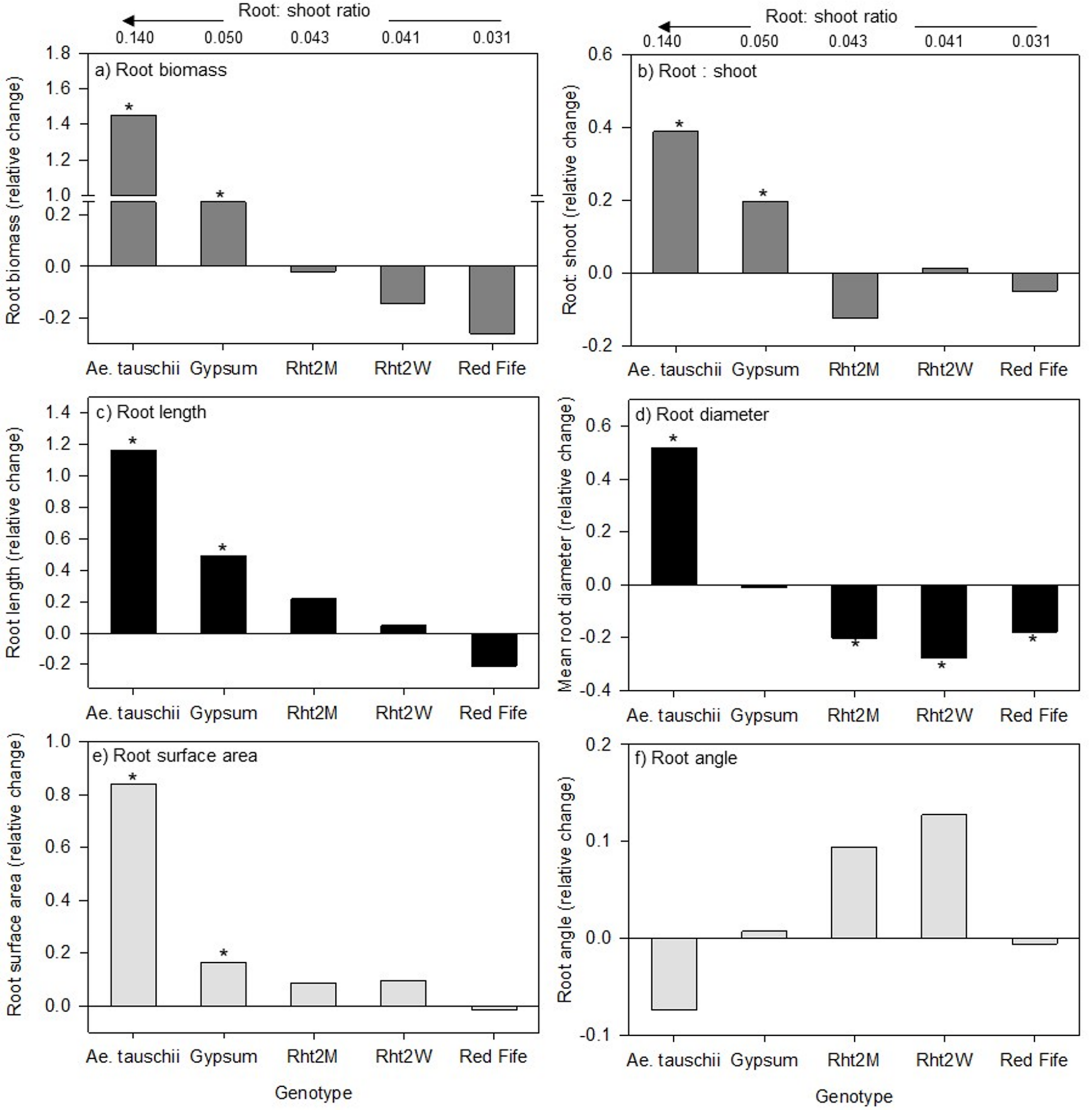
Genotypic response of root growth to compost relative to control treatments without compost or earthworms. (A) root biomass, (B) root: shoot (R:S), (C) root length, (D) average root diameter, (E) root surface area, (F) and root angle. The relative change is: mean value with compost–mean value of no compost / mean value of no compost value. Genotype responses are shown in relationship to their R:S in unamended control soils. A significant response to compost (p <0.05) is indicated by an asterisk (*); *n* = 5).

**Fig 2:**
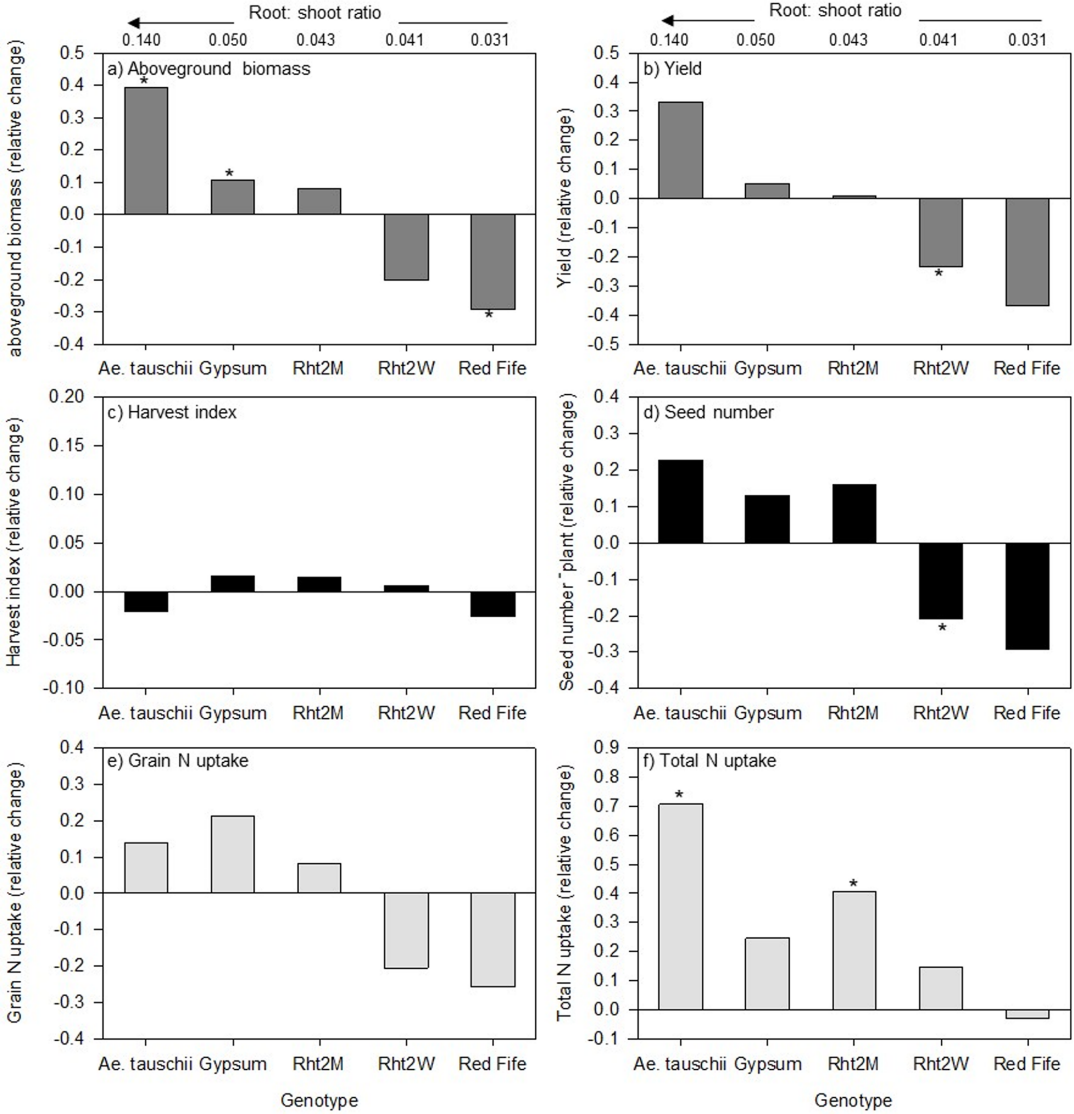
Genotypic response of aboveground growth to compost relative to control treatments without compost or earthworms. (A) aboveground biomass, (B) yield, (C) harvest index, (D) seed number, (E) grain N uptake, (F) and total aboveground N uptake. The relative change is: mean value with compost–mean value of no compost / mean value of no compost value. Genotype responses are shown in relationship to their R:S in unamended control soils. A significant response to compost (p <0.05) is indicated by an asterisk (*); *n* = 5).

Interactions between genotype and compost for all growth and biomass variables suggest that the effect of compost depends on the genotype in question (Fig 1, Fig 2). For example, genotypes with the highest R:S shoot ratios (*Ae*. *tauschii* and Gypsum) also had the greatest increase in root and aboveground biomass with compost. In *Ae*. *tauschii*, the compost treatment nearly doubled the number of tillers and increased above and belowground biomass by 40% and 158%, respectively (Table 1, Fig 1A, Fig 2A). Compost also increased R:S ratio in both *Ae*. *tauschii* (40%) and Gypsum (20%), but did not affect the remaining genotypes (Fig 1B). While no compost effect on above- or belowground biomass was observed in Rht2M and Rht2W genotypes, Red Fife, exhibiting the lowest R:S ratio in unamended soils, reduced its aboveground biomasss by 30% (Fig 2A). No other significant treatment interactions were observed for the plant growth and biomass variables.

### 3.2 Root morphology

Root image analysis indicated that genotypes differed in root length, surface areas, and average root diameter and tended to track R:S ratios (p < 0.001; Table 1). *Ae*. *tauschii* demonstrated the greatest root length per pot (476 m), approximately three to five times that observed in the *Triticum* genotypes. Gypsum had the next greatest root length (157 m), while Red Fife, Rht2M, and Rht2W showed similar root values of 88 m, 87 m, and 87 m, respectively. The average root diameter of *Ae*. *tauschii* was 1.04 mm, twice that of all wheat genotypes. Root surface area was also higher in *Ae*. *tauschii* and Gypsum by 4.5 and 1.5 times relative to the other genotypes. While root mean diameter, surface area, and length all increased with increases in R:S, difference in root angle and fine root size distribution tended to change along a genotype release date gradient. Modern genotypes exhibited the narrowest root angle whereas Red Fife had almost 1.5 times wider root branching (Table 1). In examining the relative abundances of root diameter size class, coarse roots (>2 mm) only accounted for 1% of total imaged roots by volume (S2 Fig). Fine roots were dominated by < 0.5 mm diameter roots (71%). The remainder consisted of 0.5–1 mm (25%), 1–2 mm (3%), 0.75–1.00 mm (0.4%), and > 1.00 mm (0.1%). *Ae*. *tauschii*, Gypsum and Red Fife all had similarly higher abundances of the finest roots (<0.5 mm) compared to the two modern genotypes (S2 Fig).

Compost had an overall effect on root length (p = 0.020) (Fig 1C), but not for average root diameter or root angle (Table 1, Fig 1D, Fig 1F). However, compost did influence the relative abundances of fine root classes, where <0.5 mm roots increased, and larger 1–2 mm roots decreased in abundance (S2 Fig). Earthworms had no effect on the distribution of root diameter size class. Overall, plants with compost addition had a 59% greater root length and 56% greater root surface area than plants without compost. Earthworms decreased average root diameter (0.72 mm) compared to soils without earthworms (0.80 mm; p < 0.001). The specific root length was similar across all genotypes and was not affected by either compost or earthworms alone (data not shown).

An interaction between genotype and compost was observed for root length (p = 0.002), surface area (p < 0.001), and average root diameter (p < 0.001; Table 1, Fig 1C, Fig 1D, Fig 1E). Genotypes with higher R:S ratios had the largest response in root length and diameter with compost additions. Average root diameters increased by 47% for *Ae*. *tauschii* in the presence of compost but declined as R:S ratios declined (20% decrease for Rht2M and 25% for Rht2W) (Fig 1D). Conversely, root length increased as root biomass increased in response to compost (Fig 1C). Root length of *Ae*. *tauschii* more than doubled in the presence of compost, while Gypsum, Rht2M and Rht2W showed an increase of 46%, 22%, and 8%, respectively. Meanwhile, Red Fife root length decreased by 21% (non-significant) in the presence of compost. In general, the treatments receiving compost and earthworms (C-E) exhibited the lowest root diameter (0.52 mm).

### 3.3 Yield

The modern genotypes Rht2M and Rht2W were the first to reach maturity followed by Gypsum (landrace) 10 days later. Red Fife and the wild genotype were harvested one and two months, respectively, after the modern genotypes. Genotype had significant effects on yield variables. Averaging across all treatments, modern genotypes (Rht2M and Rht2W) were highest in most yield variables except average weight per seed (Table 2). For example, the modern genotypes demonstrated higher seed yield (3.28 g plant^-1^ for Rht2M and 2.79 g plant^-1^ for Rht2W) than landrace genotypes (2.57 g plant^-1^ for Gypsum and 2.56 g plant^-1^ for Red Fife). The modern genotype Rht2M had the highest harvest index (0.33), which was not significantly different from Rht2W (0.28) and Red Fife (0.28), but higher than Gypsum (0.22). The *Ae*. *tauschii* accession had the highest number of seeds, but lower yield (total seed biomass), average seed weight, and harvest index, compared to all other genotypes.

Compost had a negative effect on average weight per seed (p = 0.035) and harvest index (p = 0.014), such that plants with compost had lower average seed weight (16.9 mg seed^-1^) and harvest index (0.23) compared to treatments without compost (18.5 mg seed^-1^ and 0.25 for harvest index). Earthworms also had a similar effect, such that the number of seeds was 18.4% lower in treatments with earthworms (p = 0.009) and the harvest index showed a marginally significant decrease of 8.5% in the presence of earthworms (p = 0.089).

Compost and earthworm interactions resulted in the lowest average seed weight (p = 0.031) and harvest index (p = 0.049; Table 2) compared to other combinations, with more than 10% and 22% lower average seed weight and harvest index. The yield (total seed weight) response to compost followed R:S ratios; beginning with a positive effect on high R:S genotypes, but becoming increasingly more negative with lower root biomass, though this was only significant for Red Fife (Fig 2B). Thus, while wheat genotypes with higher R:S had relatively poorer yields in unamended soils, they outperformed in total seed weight under compost treatments, relative to the higher-performing genotypes under no compost.

### 3.4 Plant nitrogen uptake and grain quality

Genotype demonstrated significant impacts on all plant N variables. The *Ae*. *tauschii* accession was lower for all N variables compared to wheat genotypes (Table 3). Of the wheat genotypes, without compost, Gypsum exhibited the highest vegetative, but lowest proportion of N uptake to grain (p <0.05). Compost increased total aboveground N uptake on average by 24.3% but decreased the proportion of grain N uptake relative to total aboveground N uptake by 24.7%. Earthworms lowered total N uptake and the proportion of total N in the grain on average by 7.0% and 11.8%, respectively (Table 3).

**Table 3:** Means and ANOVA results for nitrogen concentration and uptake for *Ae*. *tauschii* and four spring wheat genotypes grown in the greenhouse under differing soil treatments. Standard errors are presented to the right of each mean.

Treatment^a^^b^	Vegetative biomass N concentration (%)	Grain N concentration (%)	Vegetative biomass N uptake (mg plant^-1^)	Grain N Uptake (mg plant^-1^)	Total above-ground N uptake (mg plant^-1^)	Grain N uptake to total plant N uptake (g g^-1^)
*Ae*. *tauschii*						
NC-NE	0.49 ± 0.02	3.54 ± 0.07	56.0 ± 2.5	39.2 ± 1.3	95.1 ± 3.0	0.41 ± 0.01
NC-E	0.75 ± 0.12	3.77 ± 0.11	69.6 ± 8.0	24.2 ± 5.3	93.8 ± 4.0	0.26 ± 0.06
C-NE	0.74 ± 0.07	3.29 ± 0.23	119.0 ± 5.1	41.0 ± 9.1	160.0 ± 11.6	0.25 ± 0.04
C-E	0.89 ± 0.16	3.31 ± 0.40	135.1 ± 8.4	28.2 ± 21.4	163.2 ± 29.4	0.12 ± 0.09
Gypsum						
NC-NE	1.26 ± 0.07	4.20 ± 0.20	137.2 ± 20.8	110.8 ± 14.3	248.0 ± 33.5	0.45 ± 0.02
NC-E	1.45 ± 0.05	3.93 ± 0.27	146.8 ± 12.0	84.7 ± 21.8	223.5 ± 34.1	0.31 ± 0.06
C-NE	1.24 ± 0.14	4.20 ± 0.09	164.7 ± 30.6	141.0 ± 16.6	305.7 ± 44.8	0.47 ± 0.03
C-E	1.67 ± 0.02	4.68 ± 0.19	192.4 ± 11.5	91.5 ± 8.5	283.8 ± 19.9	0.32 ± 0.01
Rht2M						
NC-NE	1.28 ± 0.24	3.58 ± 0.27	95.0 ± 11.7	92.4 ± 17.3	187.4 ± 26.2	0.48 ± 0.04
NC-E	0.80 ± 0.12	3.44 ± 0.27	81.0 ± 9.9	126.3 ± 7.6	207.3 ± 10.9	0.61 ± 0.04
C-NE	1.64 ± 0.12	3.64 ± 0.04	165.2 ± 11.8	128.9 ± 24.4	294.1 ± 34.2	0.43 ± 0.03
C-E	1.63 ± 0.16	3.67 ± 0.17	152.4 ± 22.3	107.9 ± 25.9	260.2 ± 44.8	0.40 ± 0.04
Rht2W						
NC-NE	1.00 ± 0.08	3.68 ± 0.09	113.2 ± 11.1	126.8 ± 10.3	240.1 ± 18.5	0.53 ± 0.02
NC-E	0.86 ± 0.10	3.42 ± 0.17	73.5 ± 8.7	93.3 ± 10.0	166.8 ± 13.6	0.56 ± 0.04
C-NE	1.68 ± 0.07	3.47 ± 0.13	162.8 ± 22.8	104.7 ± 18.7	267.8 ± 38.3	0.39 ± 0.04
C-E	1.90 ± 0.31	4.50 ± 0.45	132.5 ± 24.2	73.7 ± 30.2	206.2 ± 51.2	0.30 ± 0.09
Red Fife						
NC-NE	1.00 ± 0.05	3.83 ± 0.25	100.1 ± 5.6	109.0 ± 4.2	209.1 ± 8.1	0.52 ± 0.01
NC-E	1.09 ± 0.15	3.72 ± 0.11	119.3 ± 14.6	122.6 ± 15.6	242.0 ± 17.7	0.50 ± 0.05
C-NE	1.83 ± 0.15	4.20 ± 0.03	133.8 ± 14.8	82.3 ± 12.8	216.1 ± 23.2	0.38 ± 0.03
C-E	1.84 ± 0.10	4.49 ± 0.15	132.0 ± 27.5	89.6 ± 26.4	221.5 ± 53.9	0.38 ± 0.03
G	***	***	***	***	***	***
C	***	*	***	ns	***	***
E	ns	ns	ns	ns	ns	*
G x C	***	*	*	ns	*	*
G x E	*	ns	*	ns	ns	*
C x E	ns	*	ns	ns	ns	ns
G x C x E	ns	ns	ns	ns	ns	ns

^a^ No compost or earthworms added (NC-NE); no compost, but earthworms added (NC-E); compost added, no earthworms (C-NE); both compost and earthworms added (C-E).

^b^ Significance indicated (*, p < 0.05; **, p < 0.01; ***, p < 0.001; ns, not significant) for all experimental factors and interactions (G, genotype; C, compost; E, earthworm); *n* = 5.

Significant interactions of genotype x compost (p = 0.011) and genotype x earthworm (p = 0.034) were found for N uptake in the vegetative biomass. Compost increased total aboveground N uptake, except for Red Fife (Fig 2F). The highest increases occurred in *Ae*. *tauschii* (71%), followed by modern genotypes Rht2M (40%) with intermediate R:S ratio and Gypsum (25%). Similarly, *Ae*. *tauschii* and Rht2M also showed the highest vegetative N uptake response to compost. Grain N uptake response to compost tended to increase with increasing R:S ratio, however the response was insignificant for all genotypes (Fig 2E). Earthworms decreased the vegetative biomass N uptake in Rht2W, but had no significant effect on the other genotypes (Table 3).

The proportion of grain N uptake to total aboveground N uptake decreased with compost additions for *Ae*. *tauschii* (39%), Rht2W (26%), Red Fife (26%), and Rht2M (10%), but there was no effect on Gypsum (Table 3). Among wheat genotypes, this negative compost effect on relative grain uptake became increasingly higher with lower R:S ratios. Plants with compost additions had higher grain N concentration than without compost for Gypsum (0.37%), Red Fife (0.57%), Rht2M (0.14%), and Rht2W (0.14%), while in *Ae*. *tauschii*, it was lower by 0.36%.

### 3.5 Soil nitrogen

**Fig 3:**
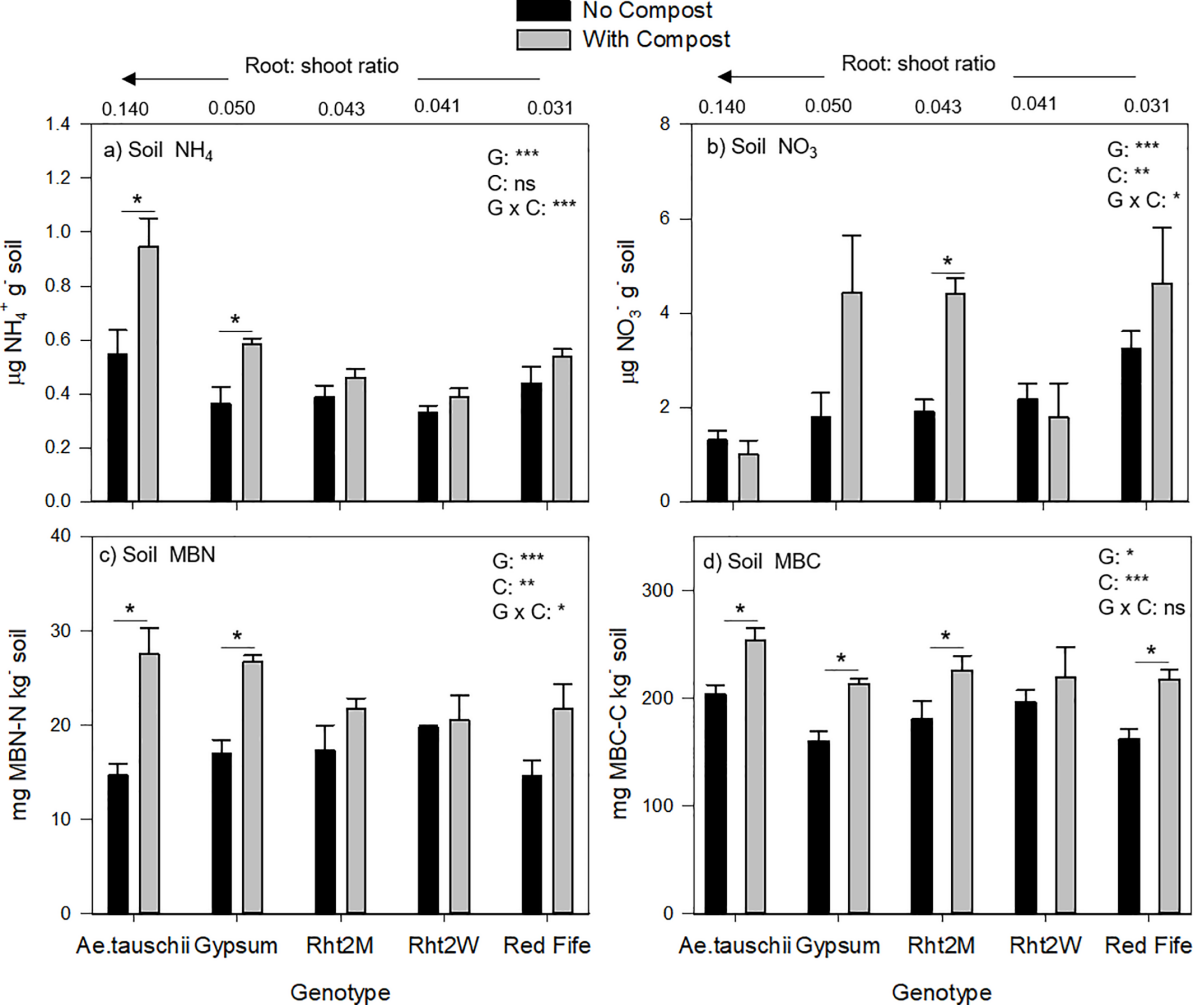
Soil N-cycling variables at wheat harvest across genotypes with and without compost additions. Soil (A) ammonium, (B) nitrate, (C) microbial biomass N, (D) and microbial biomass C concentrations are shown in relationship to genotype root:shoot (R:S) ratios in unamended soils. ANOVA result are shown for experimental factors and interactions (G, genotype; C, compost). Significant effect of compost within genotype is indicated by asterisk (*). Error bars are standard error; *n* = 5.

From wheat heading to harvest, soil NO_3_^-^ concentrations decreased while NH_4_^+^ increased, when averaged across genotype and compost (p <0.001) (S3 Fig). At harvest, we observed significant genotypic effects on all soil N-cycling and microbial biomass variables (NH_4_^+^, NO_3_^-^_,_ MBC, MBN and MB C:N) (Fig 3). Without compost, soil for all genotypes had similar NH_4_^+^ concentrations, whereas NO_3_^-^ was highest in Red Fife and lowest in *Ae*. *tauschii* soil. Compost additions had a generally positive effect on MBC, MBN and NO_3_^-^, but not on NH_4_^+^. Interactive effects between compost and genotype were observed for soil NH_4_^+^, MB C:N, and soil NO_3_^-^. Among genotypes with compost amendments, *Ae*. *tauschii* soil had almost twice as much NH_4_^+^ (0.98 μg g^-^), followed by Gypsum (0.58 μg g^-^). Specifically for NH_4_^+^ and MBN, we observed that the response to compost became increasingly more positive for genotypes with greater root biomass. *Ae*. *tauschii*, Gypsum, and Rht2M had the largest change in soil NH_4_^+^ with compost additions, while Red Fife and Rht2W exhibited no response (Fig 3A). *Ae*. *tauschii* and Gypsum also showed the greatest increase in MBN with compost compared to all other genotypes (Fig 3C). Compost also decreased MB C:N, but only for *Ae*. *tauschii* and Gypsum (data not shown).

### 3.6 Relationships among root, soil, and N uptake parameters

**Fig 4:**
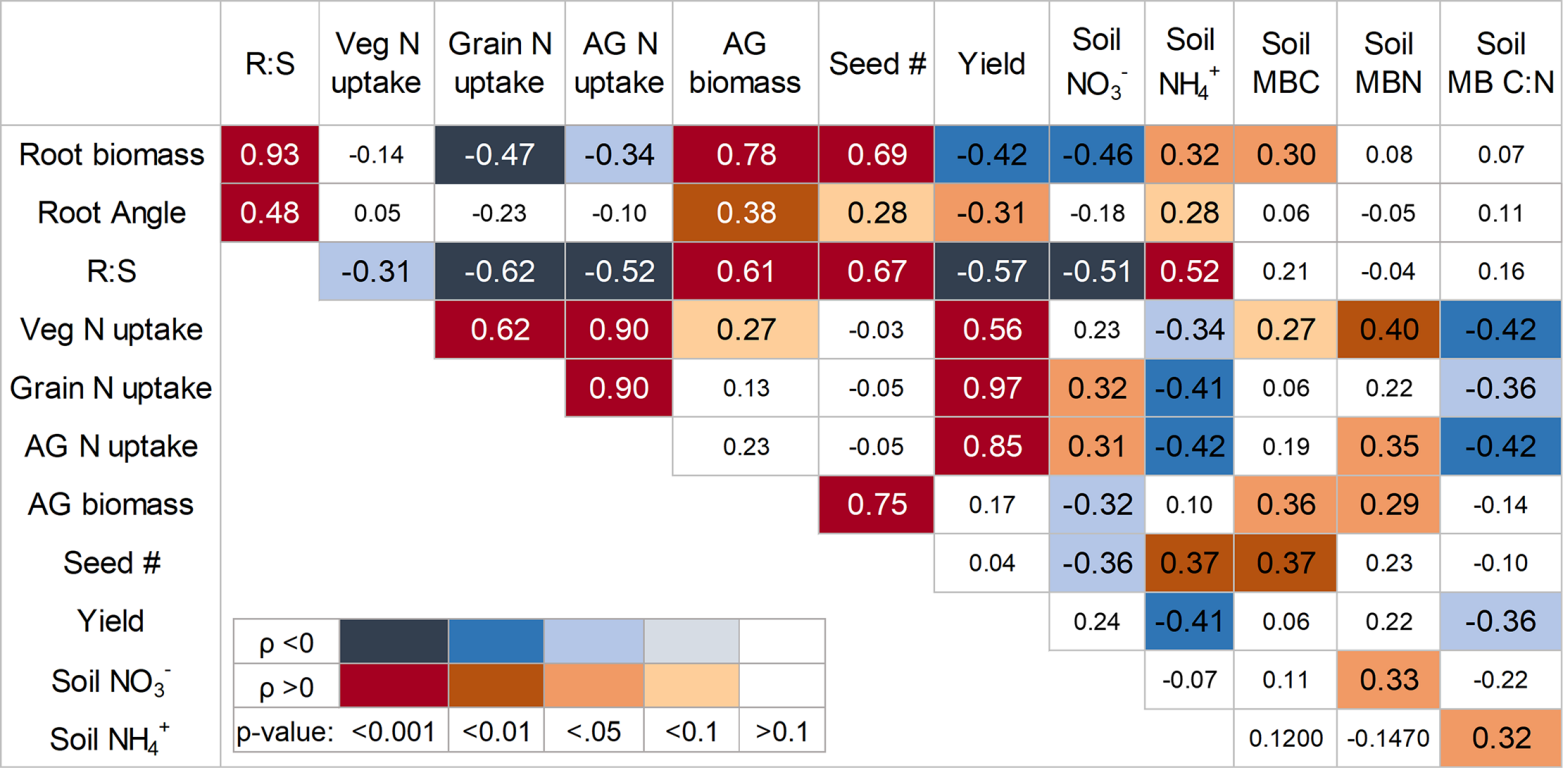
Correlations for aboveground biomass, nitrogen uptake, seed number, yield, root biomass, and soil N-cycling parameters at wheat harvest. Pearson’s correlation coefficients (ρ) are color coded by significance value and direction of correlation. Correlations are between all compost and genotype treatments (excluding earthworms) (*n* = 47). R:S is the root: shoot ratio; Veg N is the vegetative nitrogen uptake, excluding grain; AG is total aboveground biomass; Seed # is the number of seeds per plant; MBC and MBN are the concentrations of microbial biomass C and N and their ratio (MB C:N).

We compared the relationships between soil N, microbial biomass, N uptake, and root biomass from wheat harvest across all combinations of genotype and compost treatments (excluding earthworms). The highest Pearson coefficients were generally observed for R:S ratio and aboveground N and biomass parameters (ρ >0.5; p <0.001) (Fig 4). Soil parameters (NH_4_^+^, NO_3_^-^, MBC) had an overall smaller influence on plant growth (ρ <0.5; p <0.05). In terms of aboveground N and growth, belowground C allocation (root biomass and R:S) was negatively associated with N uptake and yield, but showed a positive influence on biomass production and seed number. Soil NH_4_^+^ and MB C:N were also negatively related to N uptake and yield, whereas higher NO_3_^-^ was more related to greater N uptake. Similarly, as MBC and MBN increased, so did N uptake, aboveground biomass, and seed number. Root biomass was associated negatively with soil NO_3_^-^, but positively with NH_4_^+^ and MBC (Fig 4).

## 4. Discussion

The low nutrient and organic C conditions of our soil are representative of many low input systems where plant-soil synergies have a critical role in enhancing organic N mineralization as a principle source of crop N [62]. Overall, our hypothesis that wheat genotypes with more belowground C inputs will have a greater response to improved soil biological function (achieved via addition of compost and/or earthworms) was supported, but significant effects differed by the response variable considered. In general, inherently higher root investment (as observed in the unamended soils) coincided with greater root plasticity under compost inputs. The degree and direction of the genotype response to compost generally followed a gradient of belowground root allocation, where compost effects on most growth and N-cycling variables were greater with increasing root biomass and R:S ratio. On the other hand, Red Fife, which exhibited the lowest belowground C allocation had several adverse and opposing responses to compost relative to genotypes with higher R:S ratios. The multiple interactions between genotype and soil treatments for various plant growth parameters suggest that under low fertility conditions genotypic root plasticity will be an important factor affecting agroecosystem performance following the adoption of new management regimes, such as the use of organic inputs [63].

### 4.1 Genotypic effects on plant growth

Our first objective was to evaluate selected spring wheat genotypes for phenotypic differences in both above- and belowground traits. Notably, in control soils without compost or earthworm additions, we found distinct differences in belowground C allocation in terms of both the R:S ratio and total root biomass. Of the wheat genotypes, the landraces Gypsum and Red Fife were the most divergent in root investment, with Gypsum exhibiting root characteristics more similar to the wild accession. However, in the absence of compost or earthworms additions, when genotypes are arranged along this root allocation gradient, the aboveground variables do not necessarily track a root allocation gradient. For example, Gypsum exhibited the highest grain N concentration followed by Red Fife despite being on nearly opposing ends of root allocation. Only in the presence of compost did we see aboveground traits more consistently parallel belowground traits (discussed below).

It is thus likely that in unamended soils, there are other phenotypic traits besides root allocation that influence N uptake and growth. Root architecture traits such as relative abundances of fine roots, length, and branching morphology can exert strong influence on nutrient uptake and efficiency [64,65]. While the majority (>90%) of the imaged root were within the fine root classification (<2 mm), roots <0.5 mm are more associated with nutrient and water adsorption relative to their larger counterparts which would be more active in transport [66]. Narrower root angles are often coupled with deeper rooting systems whereas wider root branching, which we observed more so in the wild accession and landraces, can be associated with greater root foraging in soils closer to the surface [67,68]. While our landraces, Gypsum and Red Fife, diverged in their root biomass and R:S, they exhibited similar mean root angles and relative abundances of <0.5 mm fine roots. Consequently, it may be that some of the decoupling between root allocation and aboveground traits we observe in unamended soils could be driven by root system architecture variability.

We selected our genotypes along a historical gradient of release date based on prior evidence showing effects of domestication and breeding environments on root systems and below-ground C allocation [38,47,48]. In considering the breeding history of *T*. *aestivum* genotypes and their evolution from *Ae*. *tauschii*, we expected to see strong divergences in phenotypic traits related to root growth and morphology as well as N uptake and allocation patterns. Specifically, we anticipated and confirmed that *Ae*. *tauschii* would allocate more C towards root biomass relative to more modern genotypes (Table 1) that were bred in part for improved harvest index and under more favorable soil conditions (improved nutrient and water availability). However, root biomass allocation was not linearly related to genotype release date. The landrace Red Fife was more similar in root allocation to the modern genotype Rht2W and had the lowest root biomass and R:S ratio. Gypsum, on the other hand was more like the wild type, *Ae*. *tauschii*, both exhibiting overall greater root biomass, higher R:S ratio, root length and surface area and average root diameter, often followed by the modern Rht2M genotype (Table 1, Fig 1).

The lack of a consistent relationships between genotype release date and root biomass allocation could be a consequence of different breeding environments and selection objectives between the two landraces. Gypsum, originating from Colorado, was likely adapted to the region’s relatively arid conditions and low nutrient inputs, such that deeper root foraging and biomass allocation traits were under stronger selection. Red Fife, initially from Western Ukraine [52] was likely cultivated under a context of greater inputs and more favorable growing conditions given that Europe has traditionally invested more resources into wheat cultivation and set aside some of its most fertile soils for wheat [69].

### 4.2 Soil treatment impacts on plant growth

We expected that to promote beneficial plant-soil interactions, such as enhanced N mineralization, co-occurring changes in both plant traits and soil health need to be established. Not only would genotypes need to express traits such as greater root biomass, that could support biological communities, but soil health promoting practices, in our case compost and earthworm additions, also need to be in place to sustain a robust biological community that roots can take advantage of. In our study, both compost and earthworms yielded significant impacts on a variety of plant growth traits, regardless of genotype. However, not all traits exhibited phenotypic plasticity to the soil amendments. We recognize that phenotyping in a greenhouse removes the reality of field conditions, but standardizing the growing environment across genotypes allows us to isolate the phenotypic responses to specific changes in soil properties. For example, regardless of genotype, compost increased root length, biomass, and relative abundances of <0.5 mm roots, but had no effect on root angle or R:S ratio. On the other hand, compost resulted in only a few positive responses in aboveground growth. This may be a consequence of the low fertility conditions creating trade-offs in N allocation between yield and vegetative components [70], constraining potential aboveground phenotypic plasticity to compost. Alternatively, a more active soil biological community can sometimes result in temporarily less plant available N due to greater microbial N demands [71] which could explain the few positive aboveground responses to compost we observed. Given the relatively higher NO_3_^-^ concentrations and decreased MB C:N with compost amended soils, it appears that the microbial community was not more N-limited and thus unlikely that more N was immobilized under compost amendments.

Compost and earthworms have previously been shown to alter root traits by, for example, changing soil resource heterogeneity and soil structure [72,73]. Earthworms are known to be important regulators of soil structure and nutrient availability, and generally have positive effects on plant growth and nutrient acquisition [73]. However, observed earthworm impacts in this study were few and when they did occur, they often had a negative effect on plant growth and N uptake. For example, earthworms resulted in lower harvest index and seed number, and decreased the proportion of total N uptake allocated towards grain. These earthworm effects on plant growth and performance are unexpected, as earthworms often stimulate microbial biomass and organic matter mineralization [74,75,76]. However, given their poor survival early during wheat growth, we suspect that the earthworm effects are more a consequence of their castings rather than their activity. Earthworm castings could have reduced microbial compost mineralization by protecting compost C within rapidly formed stable microaggregates within casts [77,78]. The limited effect of earthworms in our study is largely due to their poor survival and thus inferences about their impact on wheat growth and performance should be limited.

### 4.3 Interactive effects on plant growth and performance traits

In examining the interactive effects of genotype, compost, and earthworms on phenotypic traits, we expected that genotypes with greater belowground inputs would exhibit stronger responses to compost and earthworm additions, partly due to a) increased root access to nutrients and b) greater biological mineralization of organic inputs and thus nutrient uptake [79]. Several observed interactions show that genotypes with greater root biomass (the wild accession and Gypsum) responded differently than Red Fife which had the lowest R:S ratio, especially in terms of root morphology and biomass. Root morphology can be plastic in response to many soil conditions, including nutrient status [72,80], though root plasticity may depend on the genotype [81]. Importantly, we observed some interactions suggesting that plasticity to compost amendments within our genotypes depends on how certain traits like root diameter and R:S ratio are inherently expressed in the absence of soil amendments. For example, genotypes with overall higher R:S ratio and root length also had the greatest response in their R:S ratio and length under compost additions (Table 1, Fig 1). Thus, there may be sufficient inherited genotypic variation that can be used to select for phenotypic traits that will be responsive to soil health improvements [63]. Plant functional traits are often used to predict how a plant will perform under a changing environment [82,83] and it may be that traits such as R:S could be used to better predict genotype responses to changes in soil management [84].

In this study, we found that soils with organic nutrient additions increased root length, surface area, and biomass most in *Ae*. *tauschii* and Gypsum (Fig 1), indicating that these genotypes could adapt more readily to potential increases in soil biological activities. In low input and organic based systems, more investment towards roots could enhance access to nutrients and upregulate biological nutrient mineralization [6, 81, 84]. Indeed, genotypes with greater belowground C allocation corresponded to the greatest changes in MBN and soil NH_4_^+^ with compost (Fig 3). Consequently, while Gypsum, and to some degree Rht2M, tended to have relatively lower MBN and NO_3_^-^ in unamended soils, compost shifted this trend where Gypsum showed either higher or similar MBN, NH_4_^+^, NO_3_^-^ and MBC concentrations with compost, compared to lower R:S wheat genotypes. The stimulated turnover or ‘priming’ of organic N induced from root inputs has frequently been demonstrated (*sensu* [85, 86]) and we found positive correlations between NH_4_^+^ and roots (Fig 4). If organic mineralization is stimulated under *Ae*. *tauschii* and Gypsum, it is likely due to a shift in both soil resource stoichiometry as well as overall microbial activity. This would partly explain why we observe a simultaneous decrease in MB C:N and increase in NH_4_^+^ with the higher root inputs under *Ae*. *tauschii* and Gypsum (Fig 3, Fig 4). Accordingly, NO_3_^-^ concentrations, the primary source of N for crops, were lower under higher root inputs and we also observed a corresponding 20 and 40% increase in total N uptake in response to compost with Gypsum and *Ae*. *tauschii*, respectively (Fig 3). It is possible this relationship could be attributed to greater N uptake, yet without gross nitrification rates, we are not able to determine absolute differences in NO_3_^-^ production.

The genotypes with intermediate R:S ratio (Rht2M and Rht2W) are the two-near isogenic modern genotypes and behaved similarly in root morphology and biomass allocation. They were also less consistent in their responses to compost and in general exhibited the fewest significant effects. On the other hand, Red Fife, with the lowest R:S ratio, often showed opposing responses compared to *Ae*. *tauschii* and Gypsum, whereby compost negatively impacted aboveground biomass, grain N uptake, seed weight and yield (Fig 2). While grain N concentrations were generally high, sometimes indicative of low fertility conditions [87], it increased the most in Red Fife following compost additions. It appears the genotype associated with lower root biomass, R:S ratio, and root diameter in the control (no compost, no earthworm) is also inhibited by organic inputs. It is possible that low belowground investment by Red Fife coincides with lower total biological activity and turnover, and thus more N is retained within compost under Red Fife, relative to other genotypes with greater belowground inputs. Accordingly, Red Fife was the only genotype that showed an increase in MB C:N with compost, suggesting a shift towards greater microbial N limitation, which may be related to reduced belowground investments and an associated lack of N priming by this genotype.

While greater belowground C allocation tended to favor more positive response to compost for root morphology and soil N-cycling (e.g. root length, surface area, NH_4_^+^, MBN), the aboveground responses to compost were far more muted and did not necessarily translate into significant impacts on yield. Still, both total aboveground and vegetative N uptake increased the most with higher R:S genotypes. Moreover, Gypsum and Rht2M were the lowest yielding genotypes without compost additions, but became the highest yielding genotypes following compost amendments (Table 2). Thus, even though genotypes may exhibit root traits such as higher R:S or greater length, the benefits to plant-soil feedback may not be realized unless soil management is also modified to provide an environment that the roots can leverage.

## 5. Conclusions

Findings from our work suggest that the magnitude of positive compost effects on soil N-cycling, N uptake, and rooting systems in spring wheat follows increases in genotypic belowground investment. Our genotypes with lower belowground investment exhibited less plasticity to compost amendments and when there was a response it was frequently adverse to plant growth and soil N-cycling. Notably, genotypes that performed well in unamended soils (Red Fife and Rht2W) were outperformed by genotypes with greater root investment (Gypsum and RHt2M) when soils were amended with compost. This suggests that root phenotypic plasticity, especially in terms of root biomass allocation, could be an important breeding strategy for developing genotypes with performance advantages within organic and low input agroecosystems.

Targeting phenotypic traits for greater belowground C allocation can have multiple impacts on promoting beneficial plant-soil interactions as well as overall soil health. Greater root systems likely have higher root exudation, which encourages plant-soil interactions related to disease suppression, nutrient acquisition and protection against abiotic stressors [88]. Moreover, roots are now thought to contribute more to soil C accrual and persistence relative to aboveground biomass [89,90], and deeper rooting systems are known to reduce NO_3_^-^ losses and improve water infiltration [91]. While we evaluated a limited number of genotypes and our observed responses could differ with variations in compost amount and quality, our results indicate that different genotypes can perform distinctly in conditions of improved soil health. Thus, there is potential to leverage root phenotypic diversity, in conjunction with improvements to soil health to facilitate beneficial plant-soil interactions. We propose that greater exploration of belowground traits could help reinstate the benefits of plant-soil interactions under conditions of improving soil health.

## Supporting information

S1 Table Minimal raw dataset for all measured plant and soil variables. (XLSX)

S1 Fig Means and ANOVA results for plant growth. Means and ANOVA results for heading date (a) and tiller production (b) for *Ae*. *tauschii* and four spring wheat genotypes grown in the greenhouse under differing soil treatments (NC-NE: no compost or earthworms added; NC-E: no compost, but earthworms added; C-NE: compost added, no earthworms; C-E: both compost and earthworms). ANOVA source significance indicated (*, p < 0.05; **, p < 0.01; ***, p < 0.001; ns, not significant) for all experimental factors and interactions (G, genotype; C, compost; E, earthworm). Error bars are standard errors (n = 5).
(PDF)

S2 Fig Distribution of fine root relative abundances. Distribution of fine root relative abundance by their total root volume (cm^3^) separated by diameter size classes 0–0.5 mm (a), 0.5–1 mm (b) and 1–2 mm (c) for the five genotypes with and without compost averaged across Earthworm treatments. Genotypes with a letter in common are not significantly different within a compost treatment and diameter size class. ANOVA results for experimental factors and interactions (G, genotype; C, compost; E, earthworm) are shown for each dimeter size class (*, p < 0.05; **, p < 0.01; ***, p < 0.001; ns, not significant). Error bars are standard error; *n* = 10.
(PDF)

S3 Fig Soil nitrogen during wheat growth. Changes in soil nitrate (a) and ammonium (b) during wheat growth averaged across genotypes and compost treatments. Growth stages with a letter in common are not significantly different. Error bars are standard error; *n* = 50.
(PDF)

## References

[1] Powlson DS, Gregory PJ, Whalley WR, Quinton JN, Hopkins DW, Whitmore AP, et al Soil management in relation to sustainable agriculture and ecosystem services. Food Pol. 2011;36: S72–S87.

[2] Grayston SJ, Wang S, Campbell CD, Edwards AC. Selective influence of plant species on microbial diversity in the rhizosphere. Soil Biol Biochem. 1998;30: 369–378.

[3] Marschner P, Yang CH, Lieberei R, Crowley DE. Soil and plant specific effects on bacterial community composition in the rhizosphere. Soil Biol Biochem. 2001;33: 1437–1445.

[4] Bais HP, Weir TL, Perry LG, Gilroy S, Vivanco JM. The role of root exudates in rhizosphere interactions with plants and other organisms. Annu Rev Plant Biol. 2006;57: 233–266. 10.1146/annurev.arplant.57.032905.105159 16669762

[5] Chaparro JM, Sheflin AM, Manter DK, Vivanco JM. Manipulating the soil microbiome to increase soil health and plant fertility. Biol Fert Soil. 2012;48: 1–11.

[6] Schmidt JE, Bowles TM, Gaudin AC. Using ancient traits to convert soil health into crop yield: impact of selection on maize root and rhizosphere function. Front Plant Sci. 2016;7: 1–11. 10.3389/fpls.2016.00001 27066028 PMC4811947

[7] Bardgett RD, Mommer L, De Vries FT. Going underground: root traits as drivers of ecosystem processes. Trends Ecol Evol. 2014;29: 692–699. 10.1016/j.tree.2014.10.006 25459399

[8] Verbon EH, Liberman LM. Beneficial Microbes Affect Endogenous Mechanisms 712 Controlling Root Development. Trends Plant Sci. 2016;21: 218–229. 10.1016/j.tplants.2016.01.013 26875056 PMC4772406

[9] de Vries FT, Bardgett RD. Plant–microbial linkages and ecosystem nitrogen retention: lessons for sustainable agriculture. Front Ecol Environ. 2012;10: 425–432.

[10] de Graaff MA, Six J, Jastrow JD, Schadt CW, Wullschleger SD. Variation in root architecture among switchgrass cultivars impacts root decomposition rates. Soil Biol Biochem. 2013;58: 198–206.

[11] de Vries FT, Wallenstein MD. Below‐ground connections underlying above‐ground food production: a framework for optimising ecological connections in the rhizosphere. J Ecol. 2017;105: 913–920.

[12] Haichar ZF, Marol C, Berge O, Rangel-Castro JI, Prosser JI, Balesdent J, et al Plant host habitat and root exudates shape soil bacterial community structure. ISME J. 2008;2: 1221 10.1038/ismej.2008.80 18754043

[13] Micallef SA, Shiaris MP, Colón-Carmona A. Influence of Arabidopsis thaliana accessions on rhizobacterial communities and natural variation in root exudates. J Exp Bot. 2009;60: 1729–1742. 10.1093/jxb/erp053 19342429 PMC2671628

[14] Bender SF, Wagg C, van der Heijden MGA. An underground revolution: biodiversity and soil ecological engineering for agricultural sustainability. Trends Ecol Evol. 2016;31: 440–52. 10.1016/j.tree.2016.02.016 26993667

[15] Leff JW, Lynch RC, Kane NC, Fierer N. Plant domestication and the assembly of bacterial and fungal communities associated with strains of the common sunflower, Helianthus annuus. New Phytol. 2017;214: 412–423. 10.1111/nph.14323 27879004

[16] Cassman KG. Ecological intensification of cereal production systems: yield potential, soil quality, and precision agriculture. Proc Natl Acad Sci USA. 1999;96: 5952–9. 10339523 10.1073/pnas.96.11.5952 PMC34211

[17] Paustian K, Lehmann J, Ogle S, Reay D, Robertson GP, Smith P. Climate-smart soils. Nature. 2016;532: 49 10.1038/nature17174 27078564

[18] Mulvaney RL, Khan SA, Ellsworth TR. Synthetic nitrogen fertilizers deplete soil nitrogen: a global dilemma for sustainable cereal production. J Environ Qual. 2009;38: 2295–2314. 10.2134/jeq2008.0527 19875786

[19] Willer H, Lernoud J. The world of organic agriculture Statistics and emerging trends 2016. Research Institute of Organic Agriculture (FiBL), Frick, and IFOAM-Organics International, Bonn; 2016.

[20] Seufert V, Ramankutty N, Foley JA. Comparing the yields of organic and conventional agriculture. Nature. 2012;485: 229–232. 10.1038/nature11069 22535250

[21] Muller A, Schader C, Scialabba NEH, Brüggemann J, Isensee A, Erb KH, et al Strategies for feeding the world more sustainably with organic agriculture. Nature Comm. 2017;8: 1290. 10.1038/s41467-017-01410-w PMC5686079 29138387

[22] Van Bueren EL, Jones SS, Tamm L, Murphy KM, Myers JR, Leifert C, et al The need to breed crop varieties suitable for organic farming, using wheat, tomato and broccoli as examples: a review. NJAS-Wageningen J Life Sci. 2011;58: 193–205.

[23] Fess TL, Kotcon JB, Benedito VA. Crop breeding for low input agriculture: a sustainable response to feed a growing world population. Sustainability. 2011;3: 1742–1772.

[24] Awad W, Byrne PF, Reid SD, Comas LH, Haley SD. Great Plains winter wheat varies for root length and diameter under drought stress. Agron J. 2018;Forthcoming (In press).

[25] Postma-Blaauw MB, de Goede RGM, Bloem J, Faber JH, Brussaard L. Soil biota community structure and abundance under agricultural intensification and extensification. Ecol. 2010;91: 460–473. 10.1890/09-0666.1 20392011

[26] Piovanelli C, Gamba C, Brandi G, Simoncini S, Batistoni E. Tillage choices affect biochemical properties in the soil profile. Soil Till Res. 2006;90: 84–92.

[27] Wu M, Qin H, Chen Z, Wu J, Wei W. Effect of long-term fertilization on bacterial composition in rice paddy soil. Biol Fert Soil. 2011;47: 397–405.

[28] Kallenbach C, Grandy AS. Controls over soil microbial biomass responses to carbon amendments in agricultural systems: A meta-analysis. Agr Ecosyst Environ. 2011;144: 241–252.

[29] Jackson LE, Bowles TM, Hodson AK, Lazcano C. Soil microbial-root and microbial-rhizosphere processes to increase nitrogen availability and retention in agroecosystems. Curr Opin Env Sust. 2012;4: 517–522.

[30] Chaudhry V, Rehman A, Mishra A, Chauhan PS, Nautiyal CS. Changes in bacterial community structure of agricultural land due to long-term organic and chemical amendments. Microb Ecol. 2012;64: 450–460. 10.1007/s00248-012-0025-y 22419103

[31] Celik I, Ortas I, Kilic S. Effects of compost, mycorrhiza, manure and fertilizer on some physical properties of a Chromoxerert soil. Soil Till Res. 2004;78: 59–67.

[32] Baldi E, Toselli M. Root growth and survivorship in cow manure and compost amended soils. Plant Soil Environ. 2013;59: 221–226.

[33] Fonte SJ, Quintero DC, Velásquez E, Lavelle P. Interactive effects of plants and earthworms on the physical stabilization of soil organic matter in aggregates. Plant Soil. 2012;359: 205–214.

[34] Araujo Y, Luizão FJ, Barros E. Effect of earthworm addition on soil nitrogen availability microbial biomass and litter decomposition in mesocosms. Biol Fert Soil. 2004;39: 146–152.

[35] Eriksen-Hamel NS, Whalen JK. Earthworms, soil mineral nitrogen and forage production in grass-based hayfields. Soil Biol Biochem. 2008;40: 1004–1010.

[36] Binet F, Fayolle L, Pussard M, Crawford JJ, Traina SJ, Tuovinen OH. Significance of earthworms in stimulating soil microbial activity. Biol Fert Soils. 1998;27: 79–84.

[37] Pérez-Jaramillo JE, Mendes R, Raaijmakers JM. Impact of plant domestication on rhizosphere microbiome assembly and functions. Plant Mol Biol. 2016; 90: 635–644. 10.1007/s11103-015-0337-7 26085172 PMC4819786

[38] Waines JG, Ehdaie B. Domestication and crop physiology: roots of green-revolution wheat. Ann Bot. 2007;100: 991–998. 10.1093/aob/mcm180 17940075 PMC2759207

[39] Milla R, Osborne CP, Turcotte MM, Violle C. Plant domestication through an ecological lens. Trends Ecol Evol. 2015;30: 463–469. 10.1016/j.tree.2015.06.006 26138385

[40] Wallenstein MD. Managing and manipulating the rhizosphere microbiome for plant health: a systems approach. Rhizosphere. 2017;3: 230–232.

[41] Kapulnik Y, Kushnir U. Growth dependency of wild, primitive and modern cultivated wheat lines on vesicular–arbuscular mycorrhiza fungi. Euphytica. 1991;56: 27–36.

[42] Hetrick BAD, Wilson GWT, Gill BS, Cox TS. Chromosome location of mycorrhizal responsive genes in wheat. Can J Bot. 1995;73: 891–897.

[43] Zhu YG, Smith SE, Barritt AR, Smith FA. Phosphorus (P) efficiencies and mycorrhizal responsiveness of old and modern wheat cultivars. Plant Soil. 2001;237: 249–255.

[44] Martín‐Robles N, Lehmann A, Seco E, Aroca R, Rillig MC, Milla R. Impacts of domestication on the arbuscular mycorrhizal symbiosis of 27 crop species. New Phytol. 2017; 10.1111/nph.14962 29281758

[45] CGIAR WHEAT. Vital Grain of Civilization and Food Security. 2013 Annual Report, CGIAR Research Program on Wheat, Mexico; 2014. Available from: http://repository.cimmyt.org/xmlui/bitstream/handle/10883/4016/99544.pdf?sequence=1 Cited June 20, 2017.

[46] Feldman M. Origin of cultivated wheat In: Bonjean AP, Angus WJ, editors. The World Wheat Book, A history of Wheat Breeding. Paris: Lavoisier Publishing; 2001 pp.3–53.

[47] Gioia T, Nagel KA, Beleggia R, Fragasso M, Ficco DB, Pieruschka R et al Impact of domestication on the phenotypic architecture of durum wheat under contrasting nitrogen fertilization. J Exp Bot. 2015;66: 5519–5530. 10.1093/jxb/erv289 26071535

[48] Iannucci A, Fragasso M, Beleggia R, Nigro F, Papa R. Evolution of the crop rhizosphere: impact of domestication on root exudates in tetraploid wheat (Triticum turgidum L.). Front Plant Sci, 2017; 8. 10.3389/fpls.2017.02124 PMC5733359 29326736

[49] Genesys, Accession profile: TA2374. https://www.genesys-pgr.org/1/acn/id/4461690 Cited January 02, 2017.

[50] Clark JA, Martin JH, Leighty CE. The common white wheat. Farmers’ Bulletin. Issues. 1922;1301–1325: 3–42.

[51] Morris CF, Lillemo M, Simeone MC, Giroux MJ, Babb SL, Kidwell KK. Prevalence of puroindoline grain hardness genotypes among historically significant North American spring and winter wheats. Crop Sci. 2001;41: 218–228.

[52] Symko S. From a single seed: Tracing the Marquis wheat success story in Canada to its roots in the Ukraine. 2002. Available from: www.agt.gc.ca. Cited April 10, 2017.

[53] Lanning SP, Bowman HF, Habernicht D, Carlson GR, Eckhoff JL, Kushnak GD, et al Registration of ‘Scholar' Wheat. Crop Sci. 2000; 40: 861–861.

[54] Lanning SP, Martin JM, Stougaard RN, Guillen-Portal FR, Blake NK, Sherman JD, et al Evaluation of near-isogenic lines for three height-reducing genes in hard red spring wheat. Crop Sci. 2012;52: 1145–1152.

[55] Schultz MJ. Soil ecological interactions of spotted knapweed and native plant species. Ph.D. Dissertation. Colorado State University, Fort Collins. 2008. Available from: https://www.semanticscholar.org/paper/Thesis-Soil-Ecological-Interactions-of-Spotted-Kna-Schultz-Paschke/d6c28903860b07bf52ed9b40918a23432ac0611a

[56] Halvorson AD, Stewart CE, 2015. Stover removal affects no-till irrigated corn yields, soil carbon, and nitrogen. Agron. J. 2015;107: 1504–1512.

[57] Reynolds JW, Damoff GA. More earthworms (Oligochaeta: Lumbricidae and Sparganophilidae) from Colorado, USA. Megadrilogica 2011;14: 159–172.

[58] Becker SR, Byrne PF, Reid SD, Bauerle WL, McKay JK, Haley SD. Root traits contributing to drought tolerance of synthetic hexaploid wheat in a greenhouse study. Euphytica. 2016;207: 213–224.

[59] Costa C, Dwyer LM, Hamel C, Muamba DF, Wang XL, Nantais L, et al Root contrast enhancement for measurement with optical scanner-based image analysis. Can J Botany. 2001;79: 23–29.

[60] Zadoks JC, Chang TT, Konzak CF. A decimal code for the growth stages of cereals. Weed Research. 1974;14: 415–421.

[61] Wu J, Joergensen RG, Pommerening B, Chaussod R, Brookes PC. Measurement of soil microbial biomass C by fumigation-extraction—an automated procedure. Soil Biol Biochem. 1990;22: 1167–1169.

[62] Gardner JB, Drinkwater LE. 2009. The fate of nitrogen in grain cropping systems: a meta-analysis of 15N field experiments. Ecol. App. 2009; 19: 2167–2184. 10.1890/08-1122.1 20014586

[63] Jain N, Singh GP, Yadav R, Pandey R, Ramya P, Shine MB, et al 2014. Root trait characteristics and genotypic response in wheat under different water regimes. Cereal Res Commun. 2014;42: 426–438.

[64] Sattelmacher B, Horst WJ, Becker HC. Factors that contribute to genetic variation for nutrient efficiency of crop plants. J Plant Nutr Soil Sci. 1994;157: 215–224.

[65] Paez-Garcia A, Motes CM, Scheible WR, Chen R, Blancaflor EB, Monteros MJ. Root traits and phenotyping strategies for plant improvement. Plant. 2015;4: 334–355. 10.3390/plants4020334 PMC4844329 27135332

[66] McCormack ML, Dickie IA, Eissenstat DM, Fahey TJ, Fernandez CW, Guo D, et al Redefining fine roots improves understanding of below‐ground contributions to terrestrial biosphere processes. New Phytol. 2015;207: 505–518. 10.1111/nph.13363 25756288

[67] Manschadi AM, Hammer GL, Christopher JT, DeVoil P. Genotypic variation in seedling root architectural traits and implications for drought adaptation in wheat (Triticum aestivum L. Plant Soil. 2008;303: 115–129.

[68] An H, Dong H, Wu T, Wang Y, Xu X, Zhang X, et al Root growth angle: An important trait that influences the deep rooting of apple rootstocks. Sci Hortic. 2017;216: 256–263.

[69] Litvinenko M, Lyfenko S, Poperelya F, Babajants L, Palamatchuk A. Ukrainian wheat pool In: Bonjean AP, Angus WJ, editors. The World Wheat Book, A history of wheat breeding. Paris: Lavoisier Publishing; 2001 pp. 351–375.

[70] Reich PB. 2014. The world‐wide ‘fast–slow’plant economics spectrum: a traits manifesto. J. Ecol. 2014; 102: 275–301.

[71] Inselsbacher E, Umana NHN, Stange FC, Gorfer M, Schüller E, Ripka K, et al 2010. Short-term competition between crop plants and soil microbes for inorganic N fertilizer. Soil Biol. Biochem. 2010; 42:360–372.

[72] López-Bucio J, Cruz-Ramırez A, Herrera-Estrella L. The role of nutrient availability in regulating root architecture. Curr Opin Plant Biol. 2003;6: 280–287. 12753979 10.1016/s1369-5266(03)00035-9

[73] Van Groenigen JW, Lubbers IM, Vos HM, Brown GG, De Deyn GB, van Groenigen KJ. Earthworms increase plant production: a meta-analysis. Sci Rep. 2014;4: 6365 10.1038/srep06365 25219785 PMC5376159

[74] Brown GG, Barois I, Lavelle P. Regulation of soil organic matter dynamics and microbial activity in the drilosphere and the role of interactions with other edaphic functional domains. Eur J Soil Biol. 2000;36: 177–198.

[75] Speratti AB, Whalen JK. Carbon dioxide and nitrous oxide fluxes from soil as influenced by anecic and endogeic earthworms. Appl Soil Ecol. 2008;38: 27–33.

[76] Bernard L, Chapuis-Lardy L, Razafimbelo T, Razafindrakoto M, Pablo AL, Legname E., et al Endogeic earthworms shape bacterial functional communities and affect organic matter mineralization in a tropical soil. ISME J. 2012;6: 213 10.1038/ismej.2011.87 21753801 PMC3246243

[77] Bossuyt H, Six J, Hendrix PF. Rapid incorporation of carbon from fresh residues into newly formed stable microaggregates within earthworm casts. Eur J Soil Sci. 2004;55: 393–399.

[78] Fonte SJ, Kong AYY, van Kessel C, Hendrix PF and Six J. Influence of earthworm activity on aggregate-associated carbon and nitrogen dynamics differs with agroecosystem management. Soil Biol Biochem. 2007;39: 1014–1022.

[79] Ming N, Pendall E. Do rhizosphere priming effects enhance plant nitrogen uptake under elevated CO_2_? Agr Ecosyst Environ. 2016;224: 50–55.

[80] Grossman JD, Rice KJ. Evolution of root plasticity responses to variation in soil nutrient distribution and concentration. Evol Appl. 2012;5: 850–857. 10.1111/j.1752-4571.2012.00263.x 23346229 PMC3552402

[81] Gaudin A, McClymont SA, Raizada MN. The nitrogen adaptation strategy of the wild teosinte ancestor of modern maize, subsp. Parviglumis. Crop Sci. 2011;51: 2780–2795.

[82] Soudzilovskaia NA, Elumeeva TG, Onipchenko VG et al Functional traits predict relationship between plant abundance dynamics and long-term climate warming. P Natl A Sci. 2013;110: 18180–18184. 10.1073/pnas.1310700110 PMC3831435 24145400

[83] Kimball S, Funk JL, Spasojevic MJ, Suding KN, Parker S, Goulden ML. Can functional traits predict plant community response to global change? Ecosphere. 2016;7: 12.

[84] Mason HE, Spaner D. Competitive ability of wheat in conventional and organic management systems: a review of the literature. Can J. Plant Sci. 2006;86: 333–343.

[85] Dijkstra FA, Carrillo Y, Pendall E, Morgan JA. Rhizosphere priming: a nutrient perspective. Front Microbiol. 2013;4: 216 10.3389/fmicb.2013.00216 23908649 PMC3725428

[86] Zhu B, Gutknecht JLM, Herman DJ, Keck DC, Firestone MK, Cheng W. Rhizosphere priming effects on soil carbon and nitrogen mineralization. Soil Biol Biochem. 2014;76: 183–192.

[87] Oscarson P. The strategy of the wheat plant in acclimating growth and grain production to nitrogen availability. J. Exper. Botany 2000;51:1921–1929. 11113170 10.1093/jexbot/51.352.1921

[88] Szoboszlay M, Lambers J, Chappell J, Kupper JV, Moe LA, McNear DH. Comparison of root system architecture and rhizosphere microbial communities of Balsas teosinte and domesticated corn cultivars. Soil Biol Biochem. 2015;80: 34–44.

[89] Rasse DP, Rumpel C, Dignac MF. Is soil carbon mostly root carbon? Mechanisms for a specific stabilisation. Plant Soil. 2005;269: 341–356.

[90] Kong AYY, Six J. Tracing root vs. residue carbon into soils from conventional and alternative cropping systems. Soil Sci Am J. 2010;74: 1201–1210.

[91] Kell DB. Breeding crop plants with deep roots: their role in sustainable carbon, nutrient and water sequestration. Ann Bot. 2011;108: 407–418. 10.1093/aob/mcr175 21813565 PMC3158691

