# Supplementary material for: Root traits and root biomass allocation impact how wheat genotypes respond to organic amendments and earthworms
Source: PLoS One. 2018 Jul 24;13(7):e0200646. doi: 10.1371/journal.pone.0200646 (PMC6057726; doi:10.1371/journal.pone.0200646)

S1 Figure. **Means and ANOVA results for plant growth.** Means and ANOVA results for heading date (a) and tiller production (b) for *Ae. tauschii* and four spring wheat genotypes grown in the greenhouse under differing soil treatments (NC-NE: no compost or earthworms added; NC-E: no compost, but earthworms added; C-NE: compost added, no earthworms; C-E: both compost and earthworms). ANOVA source significance indicated (*, $p < 0.05$; **, $p < 0.01$; ***, $p < 0.001$; ns, not significant) for all experimental factors and interactions (G, genotype; C, compost; E, earthworm). Error bars are standard errors (n= 5).

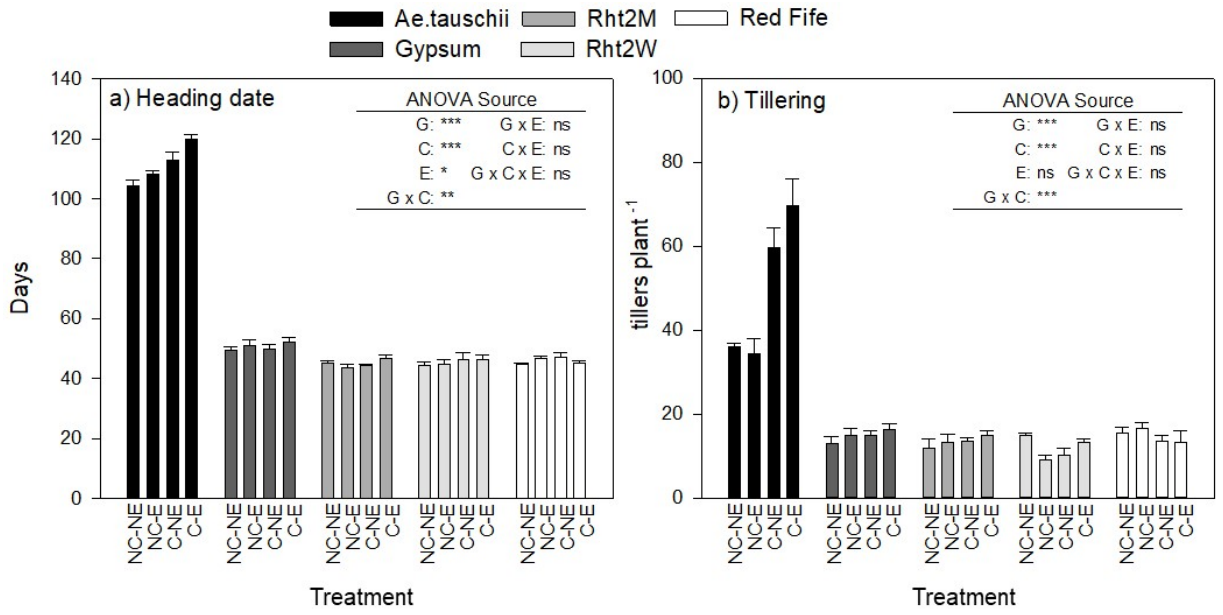

Supplement: S1 Fig — Means and ANOVA results for heading date (a) and tiller production (b) for Ae. tauschii and four spring wheat genotypes grown in the greenhouse under differing soil treatments (NC-NE: no compost or earthworms added; NC-E: no compost, but earthworms added; C-NE: compost added, no earthworms; C-E: both compost and earthworms). ANOVA source significance indicated (*, p < 0.05; **, p < 0.01; ***, p < 0.001; ns, not significant) for all experimental factors and interactions (G, genotype; C, compost; E, earthworm). Error bars are standard errors (n = 5). (PDF) [file pone.0200646.s002.pdf]
