# Supplementary material for: Root traits and root biomass allocation impact how wheat genotypes respond to organic amendments and earthworms
Source: PLoS One. 2018 Jul 24;13(7):e0200646. doi: 10.1371/journal.pone.0200646 (PMC6057726; doi:10.1371/journal.pone.0200646)

S2 Figure. Distribution of fine root relative abundances. Distribution of fine root relative abundance by their total root volume (cm^3) separated by diameter size classes 0-0.5 mm (a), 0.5-1 mm (b) and 1-2 mm (c) for the five genotypes with and without compost averaged across Earthworm treatments. Genotypes with a letter in common are not significantly different within a compost treatment and diameter size class. ANOVA results for experimental factors and interactions (G, genotype; C, compost; E, earthworm) are shown for each diameter size class (*, p < 0.05; **, p < 0.01; ***, p < 0.001; ns, not significant). Error bars are standard error; *n* =10.

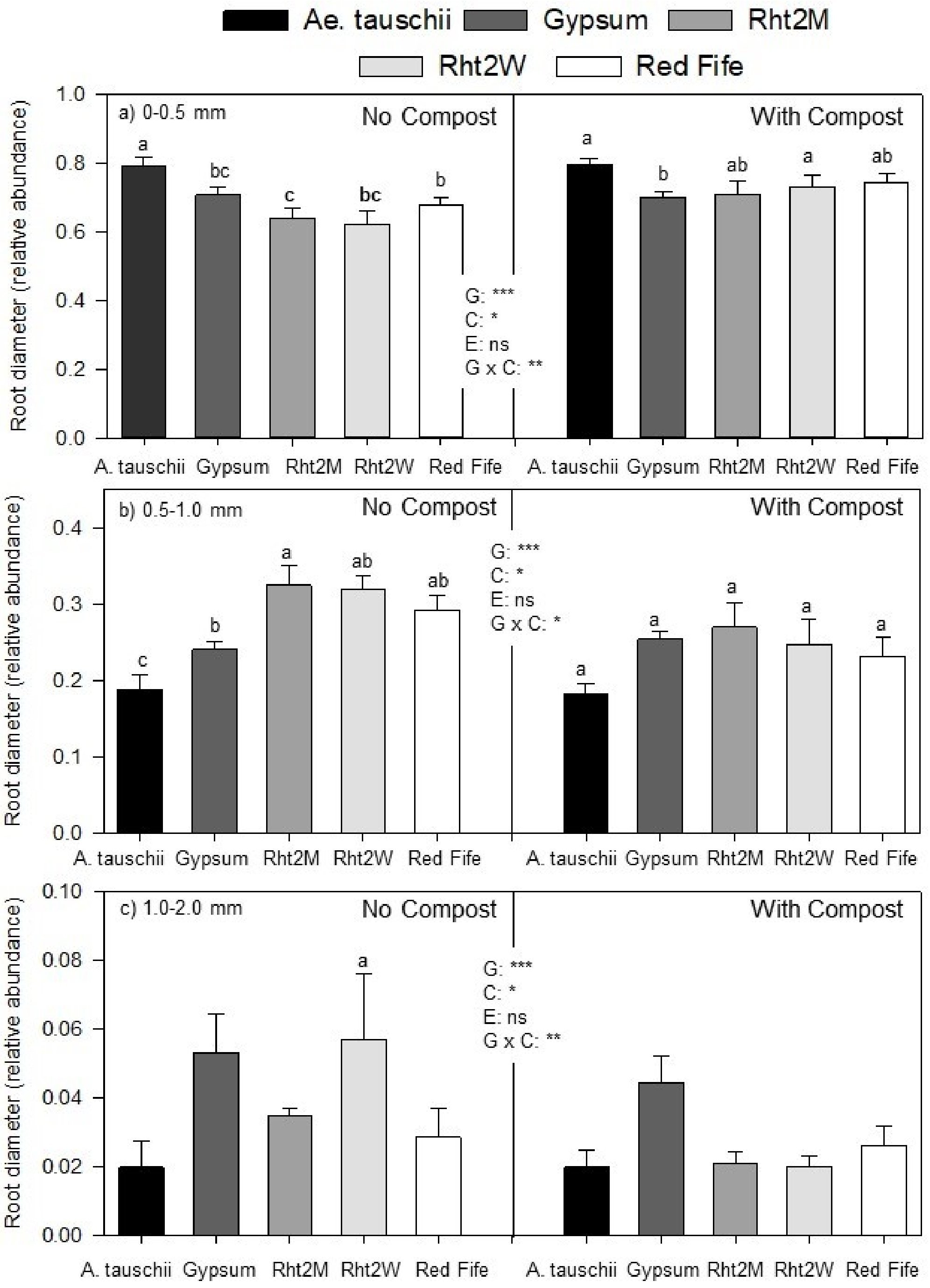

Supplement: S2 Fig — Distribution of fine root relative abundance by their total root volume (cm3) separated by diameter size classes 0–0.5 mm (a), 0.5–1 mm (b) and 1–2 mm (c) for the five genotypes with and without compost averaged across Earthworm treatments. Genotypes with a letter in common are not significantly different within a compost treatment and diameter size class. ANOVA results for experimental factors and interactions (G, genotype; C, compost; E, earthworm) are shown for each dimeter size class (*, p < 0.05; **, p < 0.01; ***, p < 0.001; ns, not significant). Error bars are standard error; n = 10. (PDF) [file pone.0200646.s003.pdf]
