# Supplementary material for: Root traits and root biomass allocation impact how wheat genotypes respond to organic amendments and earthworms
Source: PLoS One. 2018 Jul 24;13(7):e0200646. doi: 10.1371/journal.pone.0200646 (PMC6057726; doi:10.1371/journal.pone.0200646)

S3 Figure. Soil nitrogen during wheat growth. Changes in soil nitrate (a) and ammonium (b) during wheat growth averaged across genotypes and compost treatments. Growth stages with a letter in common are not significantly different. Error bars are standard error; *n*= 50.

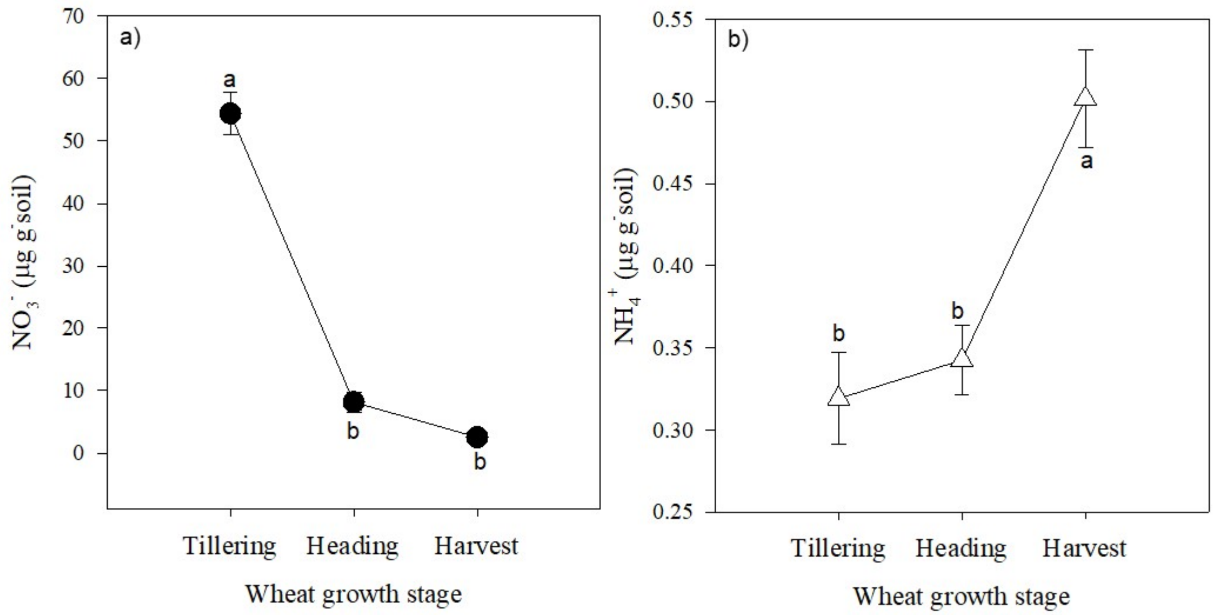

Supplement: S3 Fig — Changes in soil nitrate (a) and ammonium (b) during wheat growth averaged across genotypes and compost treatments. Growth stages with a letter in common are not significantly different. Error bars are standard error; n = 50. (PDF) [file pone.0200646.s004.pdf]
